# XAB2 depletion induces intron retention in POLR2A to impair global transcription and promote cellular senescence

**DOI:** 10.1093/nar/gkz532

**Published:** 2019-06-19

**Authors:** Shuai Hou, Dajun Qu, Yue Li, Baohui Zhu, Dapeng Liang, Xinyue Wei, Wei Tang, Qian Zhang, Jiaojiao Hao, Wei Guo, Weijie Wang, Siqi Zhao, Qi Wang, Sikandar Azam, Misbah Khan, Haidong Zhao, Liye Zhang, Haixin Lei

**Affiliations:** 1 Institute of Cancer Stem Cell, Cancer Center, Dalian Medical University, Dalian, China; 2 Breast Disease and Reconstruction Center, Breast Cancer Key Lab of Dalian, Second Affiliated Hospital, Dalian Medical University, Dalian, China; 3 School of Life Science and Technology, ShanghaiTech University, Shanghai, China

## Abstract

XAB2 is a multi-functional protein participating processes including transcription, splicing, DNA repair and mRNA export. Here, we report POLR2A, the largest catalytic subunit of RNA polymerase II, as a major target gene down-regulated after XAB2 depletion. XAB2 depletion led to severe splicing defects of POLR2A with significant intron retention. Such defects resulted in substantial loss of POLR2A at RNA and protein levels, which further impaired global transcription. Treatment of splicing inhibitor madrasin induced similar reduction of POLR2A. Screen using TMT-based quantitative proteomics identified several proteins involved in mRNA surveillance including Dom34 with elevated expression. Inhibition of translation or depletion of Dom34 rescued the expression of POLR2A by stabilizing its mRNA. Immuno-precipitation further confirmed that XAB2 associated with spliceosome components important to POLR2A expression. Domain mapping revealed that TPR motifs 2–4 and 11 of XAB2 were critical for POLR2A expression by interacting with SNW1. Finally, we showed POLR2A mediated cell senescence caused by XAB2 deficiency. Depletion of XAB2 or POLR2A induced cell senescence by up-regulation of p53 and p21, re-expression of POLR2A after XAB2 depletion alleviated cellular senescence. These data together support that XAB2 serves as a guardian of POLR2A expression to ensure global gene expression and antagonize cell senescence.

## INTRODUCTION

Gene expression is a fundamental and highly complex process that includes many steps, such as transcription, RNA splicing, RNA export, RNA degradation, translation and protein degradation (1). Regulation of gene expression is critical to a wide variety of core biological processes, such as cellular senescence (2), reprogramming (3), differentiation (4), stress responses (5), tissue homeostasis (6) and immunity (7).

In eukaryotes, the transcription of all mRNAs as well as several noncoding RNAs, including some snRNAs, snoRNAs, siRNAs and all miRNAs, is achieved by RNA polymerase II (pol II) (8). RNA pol II consists of twelve subunits in humans, while the largest and catalytic subunit is called POLR2A, also known as RPB1. The human POLR2A gene is located on chromosome 17p13.1, encoding a protein of 1970 amino acids with an apparent molecular weight of 220 kDa, and contains a C-terminal domain (CTD) of 52 heptapeptide repeats (YSPTSPS) that are essential for its polymerase activity (8–10). CTD modifications, such as phosphorylation of Ser2 and Ser5, has been studied extensively, which occur dynamically during various steps of transcription, including initiation, pausing, elongation, and termination (8–10). It is now well established that POLR2A, particularly its CTD domain, plays a key role in coordinating transcription with co-transcriptional events such as mRNA processing, thereby regulating gene expression (8–11). Therefore, POLR2A is indispensable and its loss will cause dysregulation of gene expression, leading to cell death. Furthermore, it has been reported that POLR2A is significantly down-regulated in Werner syndrome patients or old human donor cells compared with young donor cells based on microarray analysis, indicating a role in cellular senescence (12). Recurrent somatic mutations in POLR2A can drive meningiomas progression, suggesting that it may play a role in tumorigenesis (13). Despite the critical role of POLR2A in gene expression and cell function, little is known about its own regulation except for DNA damage-dependent or -independent POLR2A degradation mediated by ubiquitination (14–19).

Pre-mRNA splicing is an essential RNA processing step in eukaryotic gene expression for genes with introns. Splicing reactions take place in spliceosome, a highly dynamic macromolecular ribonucleoprotein complex composed of five snRNAs (U1, U2, U4, U5 and U6) and numerous proteins (20). The spliceosome is thought to assemble on pre-mRNAs to carry out intron excision and exon ligation in a distinctly stepwise manner. During the transition of A complex (pre-spliceosome) to B complex (pre-catalytic spliceosome), to B^act^ complex (activated spliceosome), to B* complex (catalytically activated spliceosome), to C complex (step I catalytic spliceosome), to C* complex (step II catalytically activated spliceosome), to post-splicing complex, and to the intron-lariat complex transition, a large number of proteins are involved in spliceosome assembly and activation, such as SR proteins, hnRNP proteins, Prp19-related complex, and the exon junction complex (20–23). Therefore, in addition to RNA-protein interactions, protein-protein interactions are anticipated to be widespread and play critical roles in splicing. Although specific role of many spliceosome proteins has been defined, function of many others is still elusive. These proteins are likely involved in coupling the splicing to other processes such as transcription, RNA export, or RNA quality control. As pre-mRNA splicing is important for accurate gene expression, disruption of splicing machinery will lead to multiple cellular abnormalities and human diseases (24–26).

Xeroderma pigmentosum group A (XPA)-binding protein 2 (XAB2) is a multifunctional protein involved in transcription (27,28), transcription-coupled DNA repair (27,28), homologous recombination (29), pre-mRNA splicing (28), mRNA export (30) and mitosis (31). It consists of fifteen tetratricopeptide repeat (TPR) motifs that function in protein-protein interactions and assembly of multiprotein complexes. As a member of Prp19/XAB2 complex (AQR, XAB2, Prp19, CCDC16, hISY1 and PPIE) (28) or the intron-binding complex (AQR, XAB2, CCDC16, hISY1 and PPIE) (32) or Prp19/CDC5L-related complex (33), XAB2 is identified in the human spliceosomal B (33), B^act^ (34–36), C (37), and C* (38,39) complexes required for pre-mRNA splicing. Knockdown of XAB2 in HeLa cells has been reported to inhibit Bcl-x pre-mRNA splicing (28). Previous study also revealed that XAB2 interacts with RNA polymerase II, especially the hyperphosphorylated form, and plays a role mostly in transcription elongation (27,28). Like its ortholog in human, yeast XAB2 protein SYF1 is also identified as a factor involved in pre-mRNA splicing (40) and transcription (41). In *Saccharomyces cerevisiae*, SYF1 interacts with THO complex and also mediates the interaction of Prp19 complex with RNA polymerase II via its C terminal domain, thus is recruited to transcribed genes linking transcription to mRNP formation. SYF1 mutation causes lower mRNA levels in vivo and impairs transcription in vitro, indicating that SYF1 is necessary for efficient transcription (41,42). In addition, our previous work showed that XAB2 deficiency results in mitotic cell cycle arrest by transcriptional regulation of CENPE (31), however, detailed mechanism on transcription regulation by XAB2 is still unknown.

Based on our microarray data, POLR2A mRNA was significantly down-regulated after XAB2 knockdown (31), suggesting that the impact of XAB2 on gene expression may be mediated by POLR2A. In this study, we showed that XAB2 depletion resulted in substantial loss of POLR2A at both mRNA and protein levels. However, the reduction of POLR2A was mostly due to splicing defects in POLR2A pre-mRNA caused by deficiency of XAB2. We further showed that inhibition of translation or Dom34 in mRNA surveillance pathway could at least partially rescue the expression of POLR2A, and TPR domains 2–4 and 11 of XAB2 were critical for maintaining POLR2A expression via the interaction with SNW1. Finally, we provided evidences to support that POLR2A was a major mediator of cellular senescence induced by XAB2 depletion. Taken together, our data indicate that XAB2 maintains correct splicing of POLR2A to govern gene expression and antagonize cellular senescence.

## MATERIALS AND METHODS

### Constructs and antibodies

To generate the XAB2 plasmid with HA tag in pcDNA5/FRT/TO vector (Invitrogen), previously constructed plasmid containing XAB2 cDNA (31) was used as template in PCR with primers XAB2-HA-HindIII-F and XAB2-XhoI-R, and then inserted into the vector. Full-length and truncation mutants of human XAB2 constructs resistant to siXAB2-1 were gifts from Stark Lab (29), and were re-cloned into modified pLVX-TRE3G vector (Clontech, USA) containing HA tag at the 5′ end. To generate XAB2 shRNA-1 plasmid, the oligos of XAB2-shRNA-F and XAB2-shRNA-R were annealed and then ligated into pLKO-Tet-On inducible vector (Novartis, Switzerland). POLR2A plasmid in pcDNA3.1/Hygro (+) vector containing HA tag was purchased from You Bio (China). Sequences of all the constructs were confirmed by direct sequencing. Primer sequences are listed in Supplementary Table S1.

Rabbit polyclonal antibody against XAB2 (Proteintech, 10637-1-AP, 1:800), Dom34 (Proteintech, 10582-1-AP, 1:3000), SNW1 (Proteintech, 25926-1-AP, 1:1000), SNRNP200 (Proteintech, 23875-1-AP, 1:1000), PLRG1 (Proteintech, 11914-1-AP, 1:1000), PRPF8 (Proteintech, 11171-1-AP, 1:1000), EFTUD2 (Proteintech, 10208-1-AP, 1:1000), AQR (Bethyl, A302-547A, 1:1000), CDC5L (Abcam, ab31779, 1:2000), RNPS1 (GeneTex, GTX129789, 1:1000), P53 (Proteintech, 10442-1-AP, 1:1000), Rabbit monoclonal antibody against POLR2A-CTD (Abcam, ab210527, 1:6000), POLR2A-pSer2 (Abcam, ab193468, 1:5000), POLR2A-pSer5 (Abcam, ab193467, 1:1000), CSTF2T (Abcam, ab138486, 1:1000), p21 (CST, 2947, 1:1000), mouse monoclonal antibodies against POLR2A-NTD (Santa Cruz, sc-17798, 1:800), HA tag (Biolegend, 901501, 1:1000), CPSF7 (Santa Cruz, sc-393880, 1:800), α-tubulin (Sigma, 1:8000) were used in western blots. Antibodies for ALY, Prp19, UAP56 and SC35 were gifts from Reed Lab in Harvard Medical School. Antibody against POLR2A-CTD was used in western blot for POLR2A expression in this study unless otherwise indicated. Rabbit monoclonal antibody against POLR2A (Abcam, ab210527, 1:500) was used in immunofluorescence.

### Cell culture, inhibitor treatment and RNA interference

HeLa and FRT HeLa cells were gifts from Reed Lab in Harvard Medical School, 293T, MDA-MB-231 and HFF1 cells were purchased from the American Type Culture Collection (USA). All the cells were cultured in DMEM medium supplemented with 10% FBS except for HFF1 with 15% FBS.

To inhibit pre-mRNA splicing, HeLa cells were treated with 30 uM madrasin (MCE) for 24 h. To inhibit translation, HeLa cells were treated with 50 ug/ml cycloheximide (CHX) (MCE) or 10 ug/ml emetine (Merck) for 24 h. For mRNA stability assay, HeLa cells were treated with 5 ug/ml actinomycin D (Act.D) (MCE) to inhibit transcription and then harvested at the indicated time points following addition of Act.D.

siRNA or shRNA mediated gene knockdown were performed as described before (31). All siRNAs were synthesized by RiboBio (China). Sequences of XAB2 siRNAs and shRNAs were previously reported (31). XAB2 siRNA-1 and shRNA-1 were used in XAB2 knockdown experiments in this study unless otherwise indicated. The target sequences of siRNAs are listed in Supplementary Table S2.

### Stable cell line

**Figure 1.**
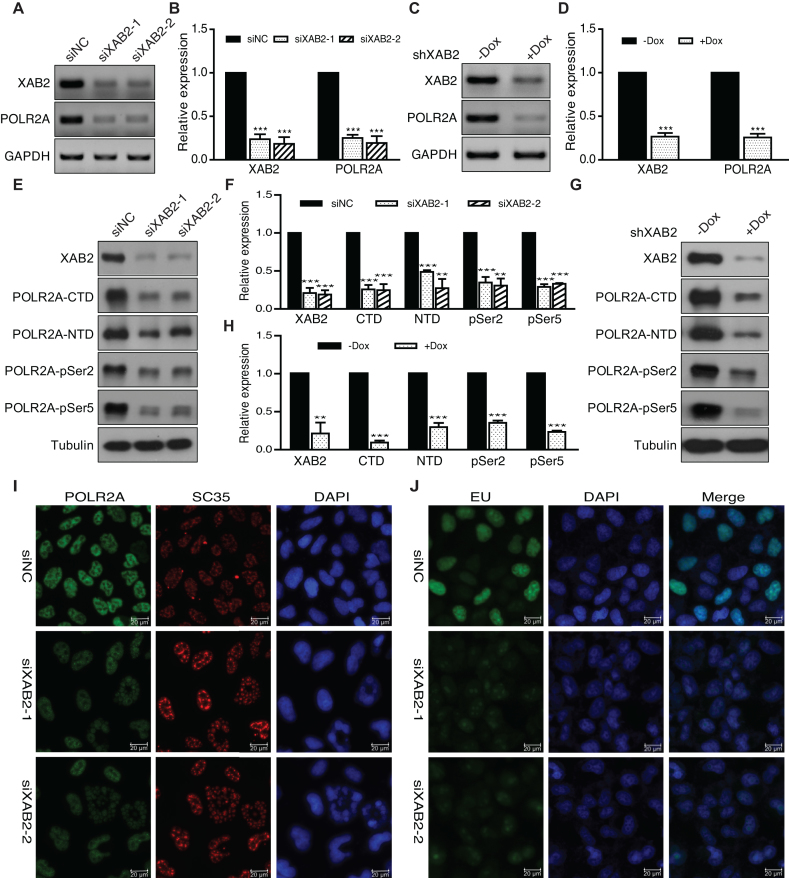
XAB2 depletion led to substantial loss of POLR2A and reduction of newly transcribed RNA. (**A**) RT-PCR showing decreased expression of POLR2A mRNA in HeLa cells treated with XAB2 siRNA. Cells were harvested after 48 hours of siRNA treatment. (**B**) Quantitation of relative mRNA expression compared to control sample in A (*n* = 3, ***P < 0.001). (**C**) RT-PCR showing decreased expression of POLR2A mRNA in HeLa cells expressing XAB2 shRNA. Cells were harvested after 72 hours of Dox induction. (**D**) Quantitation of relative mRNA expression compared to control sample in C (*n* = 3, ***P < 0.001). (**E**) Western blot showing reduction of POLR2A protein in HeLa cells treated with XAB2 siRNA. Cells were harvested after 48 h of siRNA treatment. (**F**). Quantitation of relative protein expression compared to control sample in E (*n* = 3, **P < 0.01, ***P < 0.001). (**G**) Western blot showing reduction of POLR2A protein in HeLa cells expressing XAB2 shRNA. Cells were harvested after 72 h of Dox induction. (**H**) Quantitation of relative protein expression compared to control sample in G (*n* = 3, **P < 0.01, ***P < 0.001). (**I**) Immunofluorescence (IF) staining to show substantial loss of POLR2A and altered SC35 pattern after XAB2 depletion. IF staining of POLR2A was performed after 48 h of siRNA treatment. (**J**) EU incorporation assay showing significant decrease of newly transcribed RNA after XAB2 depletion. Assay was performed after 48 h of siRNA treatment.

To establish doxorubicin (Dox)-inducible XAB2 knockdown stable cell clone used in Figure 1, XAB2-shRNA-1-pLKO-Tet-On was transfected into 293T cells together with psPAX2 and pMD2G packaging vectors at a ratio of 2:1:1. Viruses were harvested 48 h after transfection and HeLa cells were infected followed by puromycin selection.

**Figure 2.**
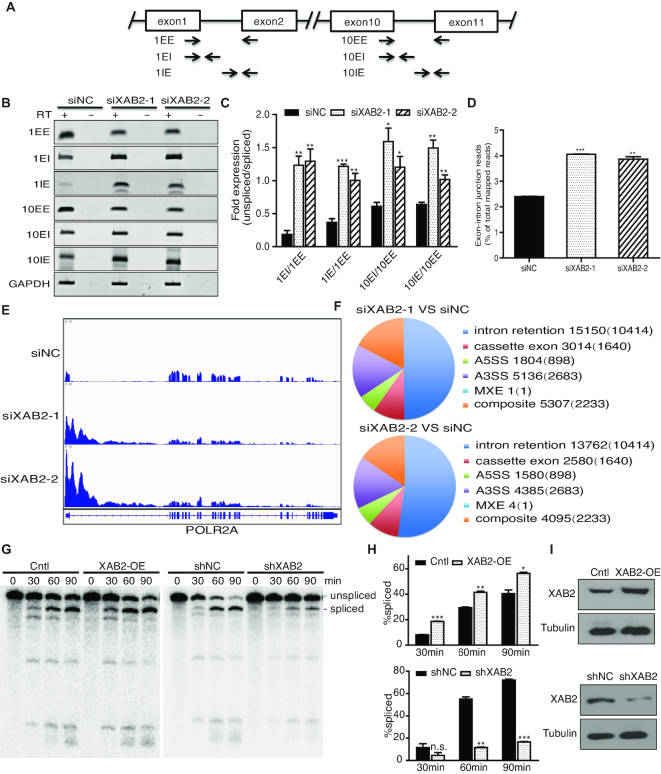
XAB2 depletion induced severe splicing defects in POLR2A. (**A**) Localization of primers using for assay in splicing efficiency. EE: both primers were in exons; EI: forward primer in exon, reverse primer in intron; IE: forward primer in intron, reverse primer in exon. (**B**) RT-PCR showing decrease of spliced POLR2A mRNA and increase of unspliced pre-mRNA after XAB2 depletion. Cells were harvested after 48 h of siRNA treatment. RT: reverse transcriptase. (**C**) Quantification showing increased ratio of unspliced vs spliced transcripts (*n* = 3, *P < 0.05, **P < 0.01, ***P < 0.001). (**D**) RNA-seq revealed much more reads mapping to exon-intron junction regions after XAB2 depletion. (**P < 0.01, ***P < 0.001). (**E**) Schematic diagram to show striking increase of reads mapping to the first exon and intron of POLR2A gene after RNA-seq. Cells were harvested after 48 hours of siRNA treatment. (**F**) Number of the categorized splicing defects after XAB2 depletion. The number in the brackets indicated the overlap between two siRNAs. (**G**) *In vitro* splicing assay showing splicing efficiency of T7-Ftz transcripts when the reactions were incubated with nuclear extract from HeLa cells over-expressing or depleting XAB2. (**H**) Quantification of splicing efficiency in G (*n* = 2, n.s.: no significance, *P < 0.05, **P < 0.01, ***P < 0.001). (**I**) Western blot to show the levels of XAB2 after over-expression or depletion.

To establish Dox-inducible XAB2 stable cell clone used in Figures 2 and 5, we used Flp-In T-Rex System based on pcDNA5/FRT/TO vector (Invitrogen) according to the manufacturer's protocol. In brief, FRT-HeLa cells were plated in 12-well plate, co-transfected with 1 ug XAB2-HA-pcDNA5/FRT/TO and 1 ug pOG44 plasmid using lipofectamine 2000, and then selected by hygromycin.

To establish Dox-inducible XAB2 full-length and truncation mutants stable cell clone used in Figure 6, lentiviruses containing pLVX-TRE3G-XAB2 and pLVX-Tet3G were produced using Rev/Gag/VSVG packaging system in 293T cells as described before (31), and transduced into HeLa cells simultaneously in a ratio of 1:1. The infected cells were selected with puromycin and G418.

### Immunofluorescence (IF)

Cells were grown in 35 mm cell culture dish with glass bottom (NEST, China), fixed with 4% paraformaldehyde (Sigma) for 30 min, followed by permeabilization with 0.5% Triton X-100 for 10 min at room temperature. Cells were then incubated with primary antibody diluted in PBS with 10% calf serum at 4°C overnight. DAPI staining was performed after incubation with Alexa-Fluor-488 or Alexa-Fluor-555-conjugated secondary antibodies (Invitrogen) diluted in the same buffer at room temperature for 30 min, images were captured using DMI8 microscope (Leica).

### Nascent RNA detection assay

Nascent RNA detection assay was performed using Cell-Light™ EU Apollo^®^488 In Vitro Imaging Kit (RiboBio, China) according to the manufacturer's protocol. Briefly, cells were grown in 35 mm cell culture dish with glass bottom (NEST, China), incubated with 500 uM ethynyl uridine (EU) in DMEM complete culture medium for 2 h. After EU labeling, cells were fixed with 4% paraformaldehyde (Sigma) for 30 min, permeabilized with 0.5% Triton X-100 for 10 min, and then stained with Apollo 488 at room temperature for 30 min, followed by DAPI staining. Fluorescence was detected using DMI8 microscope (Leica).

### RNA-seq

HeLa cells were transfected with control or XAB2 siRNA for 48 h. Total RNA was then purified using TRIzol, treated with DNase I and Ribo-Zero rRNA Removal kit to generate sequencing libraries, and subjected to strand-specific paired-end RNA-seq analyses using Illumina Hiseq X Ten platform by Novogene company (China). We used Integrative Genomics Viewer (IGV) to visualize sequence alignments (43) and junction usage model (JUM) program to identify XAB2-regulated differential alternative pre-mRNA splicing (AS) events (44). For JUM analysis, RNA-seq reads were aligned to the human reference genome hg19 using STAR 2-pass mode for more accurate junction discovery. Only unique mapped reads were considered in the downstream JUM analysis. Only splice junctions that received more than five reads in each of the replicates of the RNAi and the control knockdown samples were considered as valid junctions. IR events that received more than five reads in the upstream exon–intron and downstream intron–exon boundaries were considered as potential true IR events. A *P*-value of 0.05 was used as the statistical cutoff for differentially spliced AS events. RNA-seq data have been deposited at the NCBI Gene Expression Omnibus under the accession number GSE130087.

### *In vitro* splicing assay

T7-Ftz reporter minigene was linearized with XhoI, and pre-mRNA was synthesized using T7 RNA polymerase (New England Biolabs) in reaction mixtures containing ^32^P-UTP. Nuclear extract from XAB2 overexpression or knockdown HeLa cells was prepared as described before (45). Following incubation of ^32^P-labeled pre-mRNA with nuclear extract under splicing condition for the indicated times, RNA was purified and separated on denaturing polyacrylamide gels.

### TMT-based quantitative proteome analysis

HeLa cells were plated in six-well plate and transfected with control or XAB2 siRNA-1 for 48 h. Then cells were harvested and subjected to TMT-based quantitative proteome analysis by Jingjie PTM Biolabs (China). In brief, cell pellets were sonicated in lysis buffer (8 M urea, 1% protease inhibitor cocktail), and the extracted protein was quantified with BCA kit. Then the protein solution was digested with trypsin for two times and labeled with TMT kit (Thermo) according to the manufacturer's instructions. The tryptic peptides were fractionated into 60 fractions by high pH reverse-phase HPLC, combined into 9 fractions, and then analyzed by EASY-nLC 1000 UPLC system and Orbitrap Fusion Lumos mass spectrometer (Thermo). Raw MS files were analyzed using MaxQuant software (version 1.5.2.8). Tandem mass spectra were searched against SwissProt Human database (20,130 protein entries). TMT-6plex was specified as fixed modifications, and the false discovery rate was set at 0.01 for both protein and peptide identification. The mass spectrometry proteomics data have been deposited to the ProteomeXchange Consortium via the PRIDE (46) partner repository with the dataset identifier PXD012552.

### Protein immuno-precipitation

FRT HeLa cell line stably expressing HA-tagged XAB2 was generated using Flp-In System (Invitrogen) according to the manufacturer's protocol. To immuno-precipitate XAB2, HA antibody (Biolegend) was crosslinked to protein G Sepharose beads (GE Healthcare) with DMP. The cells were harvested 24 h after Dox induction for nucleo-cytoplasmic separation to prepare nuclear extract as previous described (45). Nuclear extract (FRT HeLa nuclear extract as negative control) was incubated under splicing condition at 30°C for 10 min in the presence or absence of ATP or RNase A. After centrifugation, the supernatant was mixed with PBS/0.1% Triton X-100 buffer and HA antibody-crosslinked beads, and rotated overnight at 4°C followed by five times washes with PBS/0.1% Triton X-100 buffer. Proteins were then eluted with protein gel loading buffer and separated on SDS-PAGE, followed by silver staining or western blot. For mass spectrometry, total proteins were precipitated with TCA.

### Gene enrichment analysis

For gene list enrichment (Gene Ontology and KEGG) analysis, genes were applied to Enrichr web application (http://amp.pharm.mssm.edu/Enrichr/) (47,48).

### SA-β-gal staining

Senescence-associated β-galactosidase (SA-β-gal) was detected using SA-β-gal Staining Kit (Beyotime, China) following the manufacturer's protocol. Cells were then photographed using Ti-S microscope (Nikon).

### Statistics

Data were presented as mean ± SD (standard deviation of the mean). Statistical analyses between two groups were performed using Student's *t*-test with statistical significance defined as: *P < 0.05, **P < 0.01 and ***P < 0.001.

## RESULTS

### XAB2 depletion results in POLR2A down-regulation and reduction in nascent transcripts

Previous studies indicated that XAB2 may function in transcription regulation (27,28), however, the molecular mechanism underlying remains poorly defined. From our previous microarray data (31), we surprisingly found that XAB2 depletion resulted in dramatic down-regulation of POLR2A mRNA (3.55-fold), the largest catalytic subunit of RNA polymerase II. To verify the result, expression level of POLR2A in HeLa cells was analyzed by RT-PCR after XAB2 depletion using two different siRNAs. As shown in Figure 1A and B, the treatment of XAB2 siRNAs led to significant decrease of XAB2 mRNA as well as POLR2A mRNA. Inducible stable HeLa cell line expressing XAB2 shRNA after Dox induction also showed reduced expression of XAB2 and POLR2A at RNA level (Figure 1C and D). Western blot as shown in Figure 1E–H indicated that XAB2 protein was efficiently depleted in HeLa cells, similarly the level of POLR2A protein was also largely reduced using antibodies against CTD, NTD or phosphorylated POLR2A (Figure 1E–H). Furthermore, treatment of XAB2 siRNAs or shRNAs in 293T or MDA-MB-231 cells led to down-regulation of XAB2 and POLR2A at both RNA and protein levels (Supplementary Figure S1A and B).

Next, IF staining of POLR2A was performed in XAB2 depleted HeLa cells, consistent with the decreased RNA and protein levels of POLR2A, POLR2A IF signal was substantially weakened compared to the control (Figure 1I). Interestingly, we also observed altered pattern of SC35 and DAPI staining, with some cells showing collapsed SC35 dots and nuclei, and some cells showing completely rounded and enlarged SC35 dots (Figure 1I and Supplementary Figure S2).

To further determine whether down-regulation of POLR2A after XAB2 depletion will have an impact on global transcription, we depleted XAB2 in HeLa cells and analyzed the expression of newly transcribed RNA using fluorescent labeled EU incorporation assay. As shown in Figure 1J, the level of newly transcribed RNA was reduced strikingly after depletion of XAB2. Together, these data pinpointed to a critical role of XAB2 in maintaining the RNA and protein levels of POLR2A and in transcription driven by RNA pol II.

### POLR2A mRNA decrease after XAB2 depletion is not due to reduced transcription

To investigate how XAB2 regulates POLR2A expression, we first did time points assay using Dox-inducible XAB2 knockdown stable cell clone. As shown in Supplementary Figure S3, XAB2 was depleted after 24 h of induction, and POLR2A mRNA was down-regulated at the same time point, while POLR2A protein was not significantly down-regulated until 72 h of induction, suggesting that XAB2 knockdown caused dramatic down-regulation of POLR2A mRNA first.

We next tested whether the decrease of POLR2A mRNA is regulated at transcription level. To do this, POLR2A or CENPE promoter reporter was transfected to inducible HeLa cell line expressing shRNA against XAB2, and luciferase activities were assayed after Dox induction. As shown in Supplementary Figure S4A, knockdown of XAB2 resulted in an unexpected increase of luciferase activity for both POLR2A and CENPE promoter at 60 h of induction. Whereas at 72 h of induction, the luciferase activities were lower or close to the control, suggesting that POLR2A mRNA decrease after XAB2 depletion was not due to reduced transcription.

Next, we performed total POLR2A (detected with 8WG16) ChIP on the POLR2A gene using Dox-inducible XAB2 knockdown stable cell clone at 36 h (XAB2 was depleted but POLR2A protein was not down-regulated) and 72 h (POLR2A protein was also down-regulated) of induction respectively (Supplementary Figure S4B). As shown in Supplementary Figure S4C and D, XAB2 depletion did not decrease the accumulation of total POLR2A at the POLR2A genome including 5′ region (promoter), middle region (10EI) and 3′ region (E29) at 36 or 72 h of induction. The results suggested that the recruitment of POLR2A to its own gene was not impaired at the time points tested with the level of XAB2 depletion in the assay.

To further investigate whether down-regulation of POLR2A at RNA level induced by XAB2 knockdown is due to reduced transcription or not, we examined the level of POLR2A nascent RNA transcripts after XAB2 depletion. Western blot using specific markers to each fraction indicated clean separation of chromatin from other fractions (Supplementary Figure S4E). As shown in Supplementary Figure S4F and 4G, XAB2 depletion did not impair the nascent RNA transcripts of POLR2A or its downstream gene MAPK4 at 36 h of induction, however, at 72 h of induction, POLR2A nascent RNA level was still barely changed but MAPK4 was significantly decreased. Together, these data supported that down-regulation of POLR2A mRNA induced by XAB2 depletion was not due to reduced transcription.

### XAB2 depletion induces severe splicing defects in POLR2A

Since XAB2 is a Prp19 complex component, we then tested whether down-regulation of POLR2A is due to splicing defects induced by XAB2 depletion. We designed two sets of primers to assay intron retention of POLR2A intron 1 and intron 10 (Figure 2A), as shown in Figure 2B and C, knockdown XAB2 led to a significant increase of the ratio of unspliced transcripts to spliced transcripts at intron 1 and a moderate increase at intron 10, suggesting severe splicing defects of POLR2A after XAB2 depletion.

We next performed RNA-seq to determine the impact of XAB2 depletion on global transcription and splicing. At transcriptional level, XAB2 depletion using two siRNAs led to decreased expression of a total of 5911 and 5798 (4897 overlapped) genes and increased expression of 5778 and 5298 (4179 overlapped) genes respectively (>1.5-fold, *P* < 0.05, Supplementary Tables S3 and S4). The results suggested an impact on global transcription after loss of XAB2.

Furthermore, knockdown XAB2 with two different siRNAs resulted in similar increase of reads mapping to exon-intron junction regions (from 2.4% in control to 4.1% and 3.9% respectively, Figure 2D) or intronic regions (from 28.1% in control to 43.8% and 43.1% respectively, Supplementary Figure S5A). Consistent with RT-PCR results of increased intron retention, reads mapping to many introns including the first intron of POLR2A were substantially raised (Figure 2E and Supplementary Figure S5B). In addition, we used JUM program to further analyze global AS events on XAB2 knockdown. As shown in Figure 2F and Supplementary Table S5 and S6, 30,412 significant AS events in siXAB2-1 and 26,406 significant AS events in siXAB2-2 were identified as compared to the control (*P* < 0.05). Among them, about 50% was intron retention (IR) events. Strikingly, among 15,150 and 13,762 IR events induced by siXAB2-1 or siXAB2-2, 14,691 (97.0%) and 13,362 (97.1%) were increased levels of IR respectively, five of which were verified by RT-PCR (Supplementary Figure S5C and D). These results suggested that XAB2 knockdown primarily led to intron retention.

To further confirm the general role of XAB2 in splicing, we performed in vitro splicing assay using T7-Ftz reporter system with nuclear extract from cells overexpressing XAB2 or depletion of XAB2. As shown in Figure 2G and H, 56% of the transcripts were spliced at 90 min when incubated with nuclear extracts from HeLa cells overexpressing XAB2 as compared to 38% of the control. Moreover, only 17% of the transcripts were spliced at 90 min when incubated with nuclear extracts from HeLa cells depleted of XAB2, which showed a sharp decrease as compared to 71% in the control (Figure 2G and H). The overexpression and knockdown efficiency of XAB2 were confirmed by western blot (Figure 2I). These findings indicated that XAB2 played a critical role in splicing and down-regulation of POLR2A after XAB2 depletion was mostly due to severe splicing defects in POLR2A.

### Inhibition of splicing by madrasin leads to decreased POLR2A expression and transcription similar to XAB2 depletion

**Figure 3.**
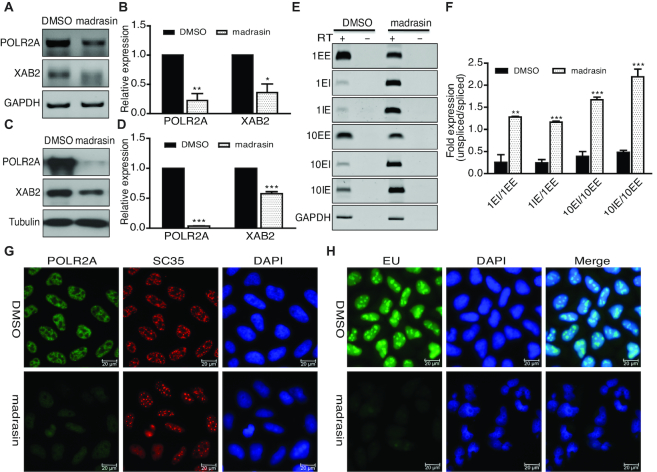
Treatment of splicing inhibitor madrasin caused reduction of POLR2A and newly transcribed RNA. (**A**) RT-PCR showing reduction of POLR2A mRNA in madrasin-treated cells. Cells were harvested after madrasin treatment (30 uM) for 24 h. (**B**) Quantitation of relative mRNA expression compared to control sample in A (*n* = 3, *P < 0.05, **P < 0.01). (**C**) Western blot showing loss of POLR2A protein in madrasin-treated cells. Cells were harvested after madrasin treatment (30 uM) for 24 h. (**D**) Quantitation of relative protein expression compared to control sample in C (*n* = 3, ***P < 0.001). (**E**) Madrasin treatment led to decrease of spliced POLR2A transcripts and accumulation of unspliced transcripts. (**F**) Quantification showing increased ratio of unspliced vs spliced transcripts (*n* = 3, **P < 0.01, ***P < 0.001). (**G**) IF staining to show loss of POLR2A protein after madrasin treatment. IF staining of POLR2A was performed after 24 h of madrasin treatment at 30 uM. (**H**) EU incorporation assay showing substantial reduction of newly transcribed RNA after madrasin treatment. Assay was performed after 24 h of madrasin treatment (30 uM).

To further determine the outcome of splicing defects in POLR2A, HeLa cells were treated with splicing inhibitor madrasin. RT-PCR and western blot revealed that POLR2A expression was down-regulated at RNA and protein levels (Figure 3A–D). The inhibitory effect of madrasin on splicing was verified by RT-PCR as the treatment led to substantial increase of unspliced transcripts (Figure 3E and F). Consistently, IF staining also supported that madrasin treatment resulted in POLR2A down-regulation (Figure 3G). However, SC35 staining was different from what was observed for XAB2 depletion, with some cells also showing rounded and enlarged SC35 dots, however some other cells showing decreased and fainted SC35 dots (Figure 3G and Supplementary Figure S6), suggesting the altered pattern of SC35 observed after XAB2 depletion was specific to the function of XAB2. In addition, nascent RNA was markedly decreased after madrasin treatment by EU incorporation assay (Figure 3H). Similarly, the expression of POLR2A in 293T or MDA-MB-231 cells was decreased at both RNA and protein levels when treated with madrasin (Supplementary Figure S7). These data together supported that splicing defects in POLR2A induced by XAB2 depletion was the major mechanism for decreased expression of POLR2A at RNA level.

### Inhibition of translation or depletion of Dom34 restores the expression of POLR2A

**Figure 4.**
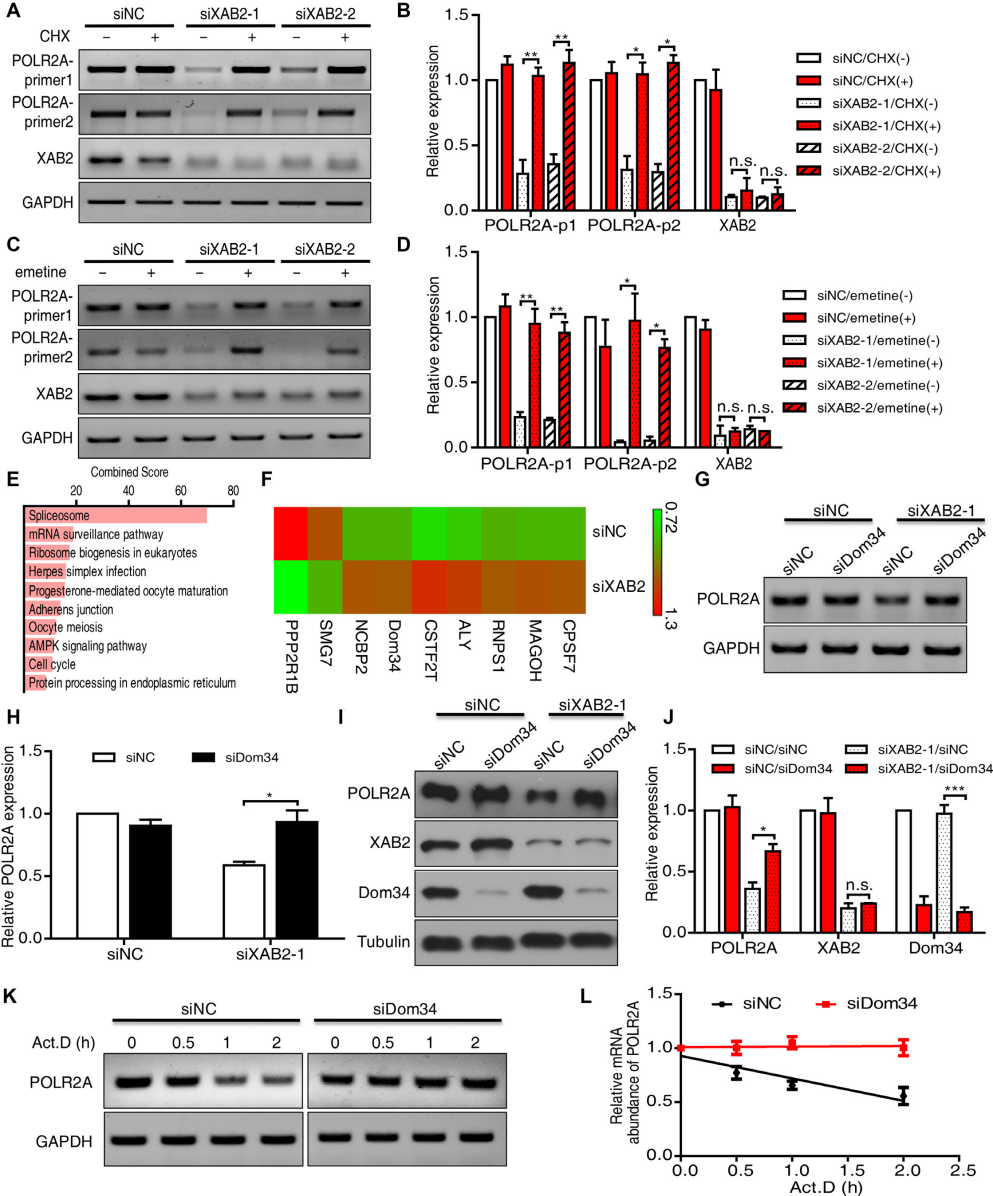
Translation and mRNA surveillance pathway were required for POLR2A RNA downregulation induced by XAB2 knockdown. (**A**) Translation inhibition by CHX rescued POLR2A at RNA level after XAB2 depletion. Cells were treated with 50 ug/ml CHX for 24 h after XAB2 depletion. (**B**) Quantitation of relative mRNA expression compared to control sample in A (*n* = 3, n.s.: no significance, *P < 0.05, **P < 0.01). (**C**) Translation inhibition by emetine rescued POLR2A at RNA level after XAB2 depletion. Cells were treated with 10 ug/ml emetine for 24 h after XAB2 depletion. (**D**) Quantitation of relative mRNA expression compared to control sample in C (*n* = 3, n.s.: no significance, *P < 0.05, **P < 0.01). (**E**) KEGG analysis based on TMT proteome results showing gene functions with significant upregulation after XAB2 knockdown. (**F**) Differential expression of genes (>1.2-fold, *P* < 0.05) related to mRNA surveillance pathway after XAB2 knockdown. (**G**) Depletion of Dom34 after XAB2 knockdown partially rescued POLR2A at RNA level. Cells were treated with siRNAs specific for XAB2 and Dom34 for 48 h. (**H**) Quantitation of relative mRNA expression compared to control sample in G (*n* = 3, *P < 0.05). (**I**) Depletion of Dom34 after XAB2 knockdown partially rescued POLR2A at protein level. Cells were treated with siRNAs specific for XAB2 and Dom34 for 48 h. (**J**) Quantitation of relative protein expression compared to control sample in I (*n* = 3, n.s.: no significance, *P < 0.05, ***P < 0.001). (**K**) Depletion of Dom34 increased the stability of POLR2A mRNA. (**L**) Quantitation of relative mRNA expression in K (*n* = 3).

To further investigate the mechanism for POLR2A mRNA decay after XAB2 depletion, we first treated XAB2-depleted HeLa cells with CHX to inhibit translation. Strikingly, RT-PCR using two sets of primers specific to POLR2A indicated that CHX treatment after XAB2 depletion fully restored the expression of POLR2A at mRNA level (Figure 4A and B). Inhibition of translation with another inhibitor emetine led to similar restoration of POLR2A mRNA (Figure 4C and D). Moreover, translation inhibition further decreased POLR2A protein level (Supplementary Figure S8). Since NMD pathway is a well-known mRNA surveillance pathway dependent on translation, we tested whether POLR2A RNA decrease caused by XAB2 knockdown depended on NMD pathway. No recovery was observed when XAB2 and UPF1, a key factor in NMD pathway, were co-knockdown (Supplementary Figure S9), suggesting other factors were involved.

In an attempt to identify such factors, we performed TMT-based quantitative proteome analysis on HeLa cells after XAB2 depletion. This analysis revealed a total of 703 proteins with significant change in expression (>1.2-fold, *P* < 0.05). Among them, 243 proteins were up-regulated and 460 proteins were down-regulated (Supplementary Table S7). KEGG analysis indicated that proteins up-regulated were mostly involved in spliceosome, mRNA surveillance pathway, and ribosome biogenesis (Figure 4E). Accordingly, a set of proteins involved in the regulation of mRNA surveillance pathway were deregulated (Figure 4F), including RNPS1, ALY and Dom34 (PELO), the expression of which were validated by western blot (Supplementary Figure S10A). Specifically, knockdown of Dom34 after XAB2 depletion resulted in partial recovery of POLR2A both at mRNA (Figure 4G and H, Supplementary Figure S10B) and protein levels (Figure 4I and J, Supplementary Figure S10C). Analysis on mRNA stability revealed that POLR2A mRNA was stabilized after Dom34 knockdown (Figure 4K and L). Together, these data suggested that inhibition of translation or depletion of Dom34 in mRNA surveillance could recover the expression of POLR2A, at least partially by stabilizing POLR2A mRNA.

### XAB2 associates with spliceosome components required for POLR2A expression

**Figure 5.**
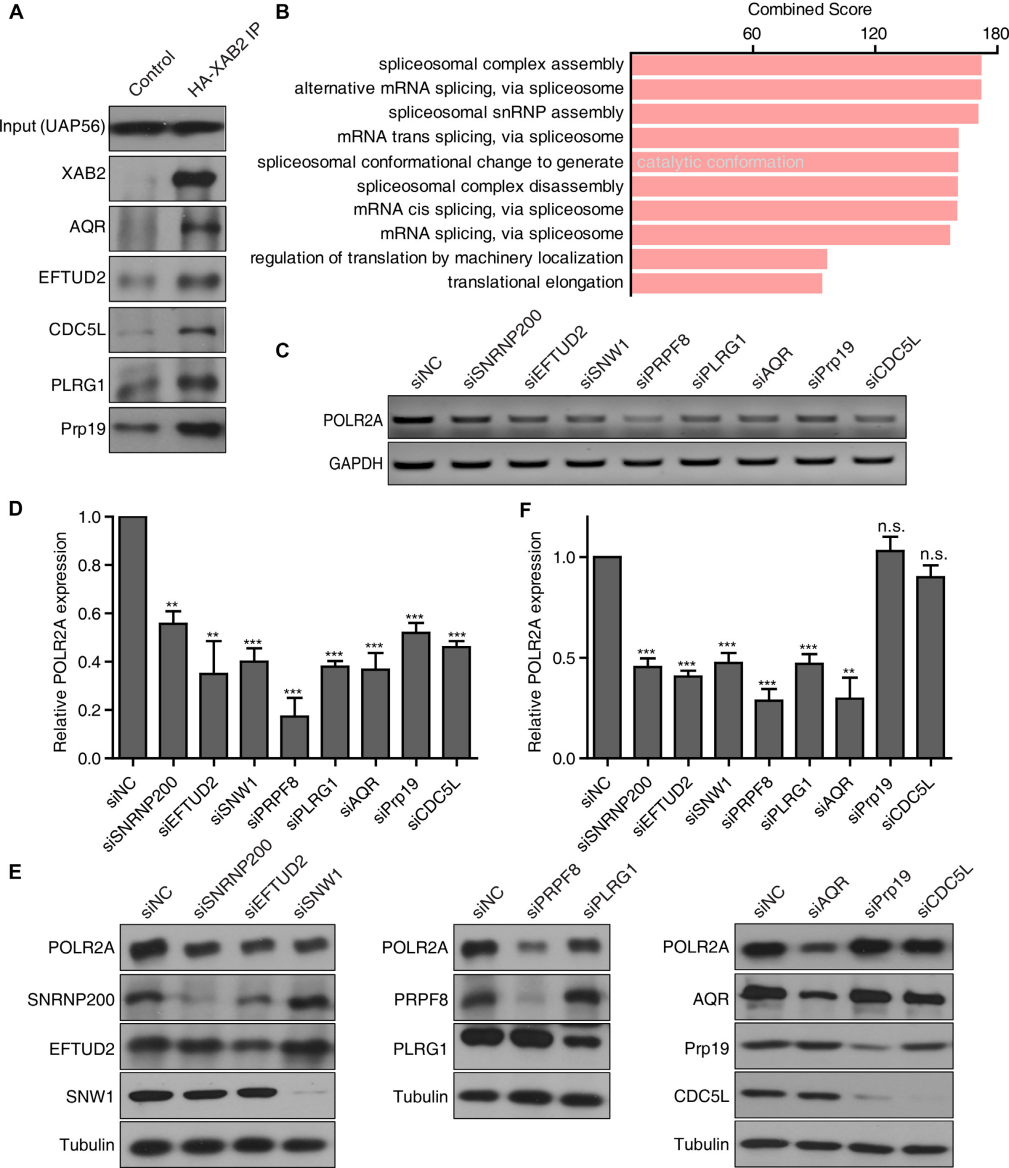
XAB2 preferentially associated with spliceosomal proteins required for POLR2A expression. (**A**) Validation of protein enrichment in XAB2 IP sample as identified by mass spectrometry analysis. (**B**) Major gene functions of XAB2 interacting proteins as revealed by Gene Ontology analysis. (**C**) Depletion of XAB2 interacting proteins impaired POLR2A mRNA expression. Cells were treated with siRNAs for 48 h. (**D**) Quantitation of relative mRNA expression compared to control sample in C (*n* = 3, **P < 0.01, ***P < 0.001). (**E**) Level of POLR2A protein after depletion of XAB2 interacting proteins. Cells were treated with siRNAs for 48 h. (**F**) Quantitation of relative protein expression compared to control sample in E (*n* = 3, n.s.: no significance, **P < 0.01, ***P < 0.001).

To identify proteins interacting with XAB2, we performed immuno-precipitation followed by mass spectrometry. Consistent with previous study (28), proteins in Prp19 complex including Prp19, CDC5L, AQR, ZNF830, PPIE and ISY1 were enriched (Supplementary Table S8). The enrichment of some of these proteins was validated by western blot (Figure 5A). Gene Ontology analysis revealed that many of these proteins were closely related to splicing (Figure 5B).

To determine if these proteins have a similar impact on POLR2A expression, we treated HeLa cells with siRNA against some of these factors. Depletion of SNRNP200, EFTUD2, SNW1, PRPF8, PLRG1 and AQR also caused a reduction at POLR2A RNA and protein levels, but depletion of Prp19 or CDC5L showed no apparent effect on POLR2A protein expression (Figure 5C–F). Together, these results indicated that XAB2 and its interacting proteins were critical for POLR2A expression.

### The TPR domains 2–4 and 11 of XAB2 have a crucial role in POLR2A expression

**Figure 6.**
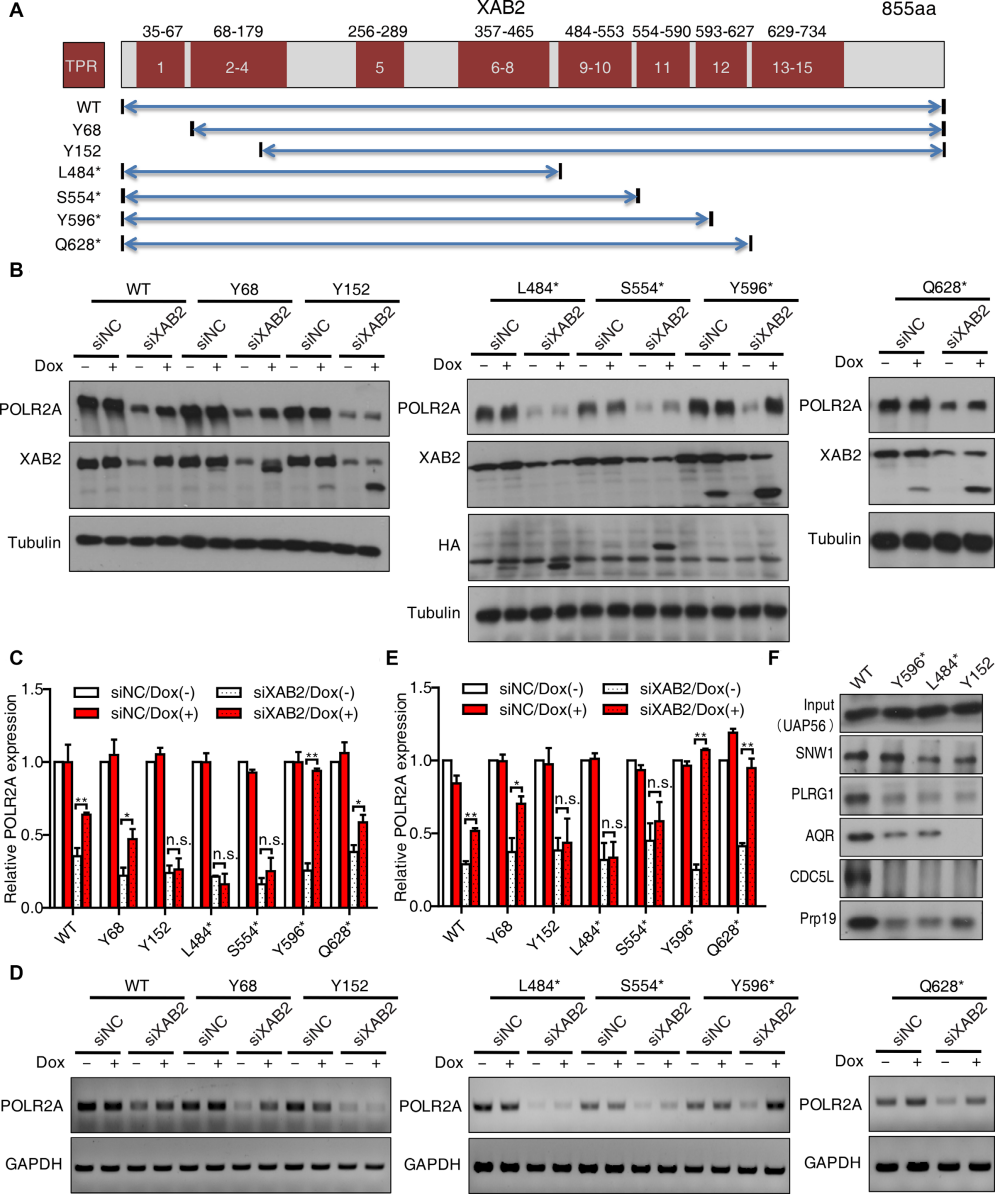
TPR domains 2–4 and 11 of XAB2 were required for POLR2A expression. (**A**) Schematic diagram of TPR domains and truncation mutants of XAB2. Numbers in red boxes indicated TPR repeats, numbers on top indicated the starting and ending positions of the amino acid for TPR repeats. (**B**) Differential capability of XAB2 truncation mutants to rescue POLR2A protein expression after XAB2 knockdown. Stable cell lines expressing XAB2 wild type or truncation mutant were induced with Dox for 48 h after XAB2 depletion. (**C**) Quantitation of relative protein expression compared to control sample in B (*n* = 3, n.s.: no significance, *P < 0.05, **P < 0.01). (**D**) RT-PCR showed differential capability of XAB2 truncation mutants to rescue POLR2A expression at RNA level after XAB2 knockdown. (**E**) Quantitation of relative mRNA expression compared to control sample in D (*n* = 3, n.s.: no significance, *P < 0.05, **P < 0.01). (**F**) Interaction of wild type XAB2 protein or truncation mutants with other proteins.

To investigate the domain requirement for XAB2-mediated POLR2A expression, we constructed Dox inducible HeLa cells stably expressing siRNA-resistant full-length human XAB2 or a series of truncation mutants (Figure 6A). Dox induction after XAB2 depletion indicated XAB2-WT, Y68 and Q628* could partially recover the expression of POLR2A (Figure 6B–E). In contrast, Y152, L484* and S554* were not sufficient to rescue POLR2A expression. Interestingly, Y596 could fully rescue POLR2A expression (Figure 6B–E). We concluded from this analysis that the TPR domains 2–4 and 11 were the core motifs necessary for XAB2-mediated POLR2A expression, similar with the core XAB2 domains function in genome maintenance (29). Next, we further examined whether these regions were required for interacting with the proteins identified in Fig 5. As shown in Figure 6F, PLRG1, AQR, Prp19 and CDC5L were only enriched in WT, however, SNW1 was enriched in WT and Y596, suggesting that the interaction of XAB2 with SNW1 via TPR domain 2–4 and 11 may be critical for POLR2A expression.

### POLR2A serves as a major mediator for cellular senescence induced by XAB2 depletion

**Figure 7.**
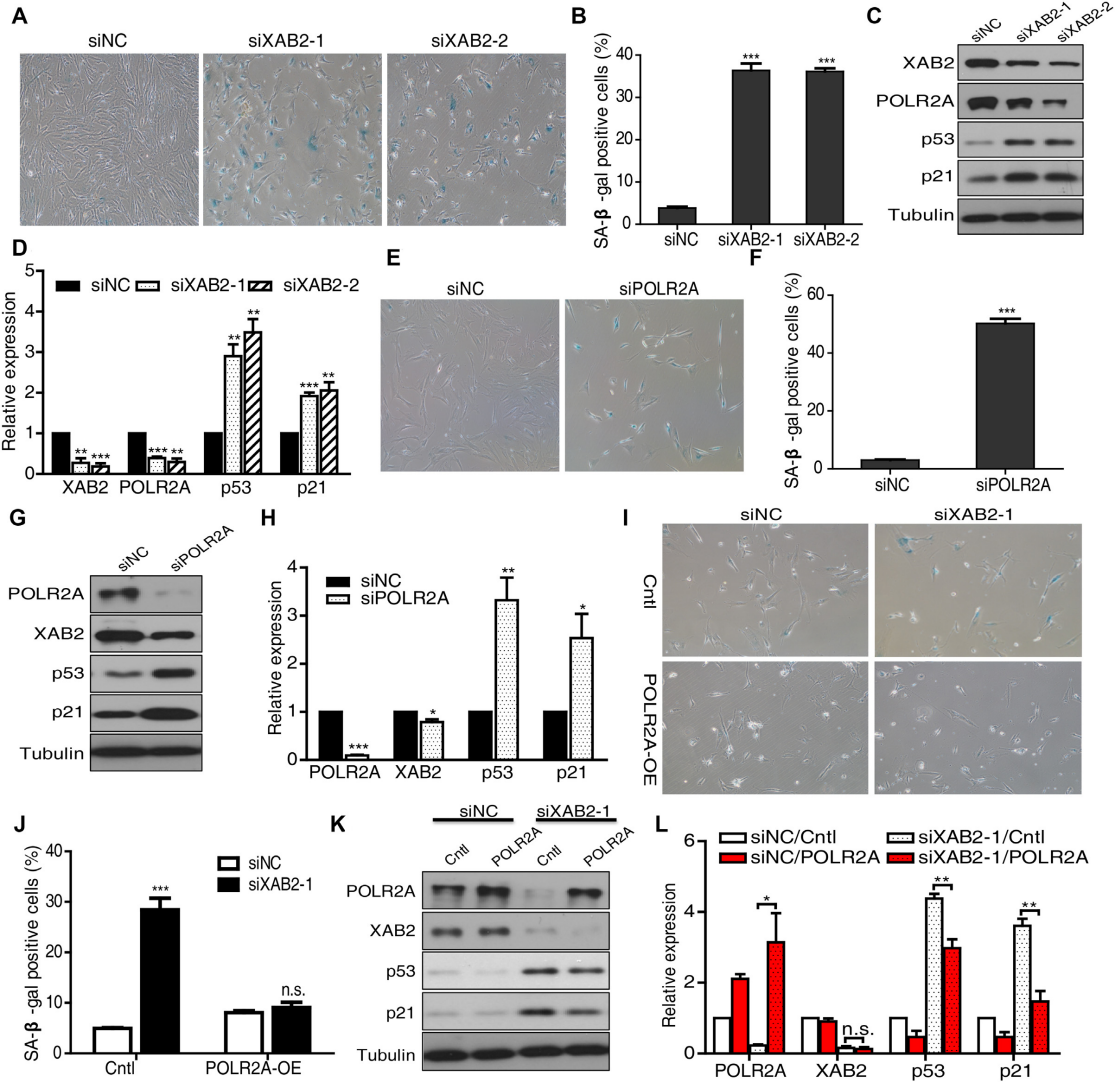
Cell senescence induced by XAB2 depletion was mediated by POLR2A. (**A**) SA-β-gal staining showed that depletion of XAB2 in HFF1 cells led to severe senescence phenotype. SA-β-gal staining was performed at 96 hours after siRNA treatment. (**B**) Significant increase of SA-β-gal positive cells after XAB2 depletion. More than 200 randomly selected cells were counted (*n* = 3, ***P < 0.001). (**C**) XAB2 depletion led to elevated expression of p53 and p21. (**D**) Quantitation of relative protein expression compared to control sample in C (*n* = 3, **P < 0.01, ***P < 0.001). (**E**) Depletion of POLR2A in HFF1 cells resulted in senescence phenotype. SA-β-gal staining was performed at 96 hours after siRNA treatment. (**F**) POLR2A depletion led to significant increase of SA-β-gal positive cells. More than 200 randomly selected cells were counted (*n* = 3, ***P < 0.001). (**G**) Depletion of POLR2A caused enhanced expression of p53 and p21. (**H**) Quantitation of relative protein expression compared to control sample in G (*n* = 3, *P < 0.05, **P < 0.01, ***P < 0.001). (**I**) Re-expression of POLR2A in XAB2 depleted HFF1 cells alleviated the senescence phenotype. SA-β-gal staining was performed at 96 hours after siRNA treatment (60 h) and POLR2A transfection (36 h). (**J**) Re-expression of POLR2A in XAB2 depleted HFF1 cells resulted in substantial reduction of SA-β-gal positive cells. More than 200 randomly selected cells were counted (*n* = 3, n.s.: no significance, ***P < 0.001). (**K**) Western blot to show that re-expression of POLR2A after XAB2 depletion led to moderate decrease of elevated p53 and p21 level. (**L**) Quantitation of relative protein expression compared to control sample in K (*n* = 3, n.s.: no significance, *P < 0.05, **P < 0.01)

To gain deep insight into the physiological role of XAB2, we checked whether XAB2 could modulate cellular senescence. First, we depleted XAB2 in the human skin fibroblast cell line HFF1, SA-β-gal staining showed that XAB2 knockdown significantly induced cellular senescence (36.4% and 36.2% in XAB2 siRNA-1 and siRNA-2 transfected cells respectively compared to 3.8% in control, *P* < 0.001, Figure 7A and B). Furthermore, XAB2 knockdown also resulted in cell cycle arrest in G2/M phase and inhibition of cell proliferation in HFF1 cells (Supplementary Figure S11A and B). Western blot on known regulators of cellular senescence including p53 and p21 indicated that XAB2 depletion led to up-regulation of p53 and p21 (Figure 7C and D).

Similarly, depletion of POLR2A in HFF1 cells resulted in striking increase of SA-β-gal positive cells (49.7% in POLR2A siRNA transfected cells compared to 3.1% in control, *P* < 0.001, Figure 7E and F). Consistent with XAB2 depletion, POLR2A knockdown led to significant increase of p53 and p21 (Figure 7G and H).

To further confirm whether XAB2 regulates cellular senescence via POLR2A, HFF1 cells were treated with XAB2 siRNA-1 followed by over-expression of POLR2A. As shown in Figure 7I and J, over-expression of POLR2A was able to recover the senescent phenotype induced by XAB2 silencing, western blot further revealed that re-expression of POLR2A resulted in moderate decrease of both p53 and p21 (Figure 7K and L), indicating that POLR2A is a major mediator of XAB2-depletion induced cell senescence.

## DISCUSSION

Gene expression is a fundamental cellular process that is essential for life. Although XAB2 has been reported to function in several regulatory processes, such as transcription, pre-mRNA splicing and mRNA export, detailed mechanism on regulation of gene expression by XAB2 remains unclear. In this study, we reported POLR2A as a major target gene down-regulated after XAB2 depletion, XAB2 depletion resulted in substantial loss of POLR2A and triggered cascades on global transcription and cell senescence.

We previously reported that knockdown XAB2 led to cell death, cell cycle blockage and mitotic defects (31), in an attempt to understand how XAB2 depletion would have such severe impact, we unexpectedly found POLR2A, the largest subunit of RNA pol II, was among the top list of genes down-regulated. We first confirmed that POLR2A was reduced at both RNA and protein levels, with RNA decreased before loss of the protein.

As SYF1, the homolog of XAB2 in yeast, has been reported to interact with RNA pol II and function in transcription elongation (41), we first tested whether decrease in POLR2A at RNA level was due to reduced transcription or not. Surprisingly, luciferase assay of XAB2 depletion showed no decrease on the transcription of POLR2A. Further assay on the enrichment of POLR2A on its own promoter indicated the recruitment of POLR2A was barely affected even when the level of POLR2A protein dropped substantially. Consistently, the nascent POLR2A RNA transcripts remained stable when nascent transcripts of MAPK4 decreased, implying the existence of specific mechanism to maintain POLR2A expression at transcription level. Overall, these evidences favored that the decrease of POLR2A mRNA was not due to reduced transcription.

Several studies reported that knockdown of proteins functioning in pre-mRNA splicing led to reduction of POLR2A. For example, depletion of splicing factor CWC22 caused POLR2A mRNA down-regulation (49), knockdown of splicing factor SF3a induced down-regulation of POLR2A protein in HeLa cells (50). In SRSF1 or SRSF2-depleted MEFs, Ser2-phosphorylated POLR2A was dramatically diminished, but the total and Ser5-phosphorylated POLR2A showed minor or no reduction (51), the same effect as observed in HeLa cells treated with pre-mRNA splicing inhibitor SSA or Pla-B (52). Consistent with these studies, our results indicated a similar reduction of POLR2A after XAB2 depletion. Furthermore, the decrease of POLR2A is explained by severe splicing defects in POLR2A caused by deficiency of XAB2. Specifically, intron retention at POLR2A intron 1 and intron 10 was detected using primer sets for unspliced pre-mRNA. RNA-seq analysis further supported that XAB2 depletion led to a wide range of splicing defects, with more than 50% events were intron retention. In addition, in vitro splicing assay also indicated XAB2 over-expression promoted splicing, whereas XAB2 depletion inhibited splicing. Based on these results, we conclude that the primary effect of XAB2 depletion is splicing defect, which also explains reduced POLR2A at RNA level at early time points.

At late time points of XAB2 depletion, POLR2A protein lost, which should have a significant impact on global transcription and indeed we observed substantial decrease of nascent transcripts using EU incorporation assay. The impact on global transcription was further supported by RNA-seq with a large number of genes showing decreased expression with the other large number of genes showing elevated expression. Consistently, treatment of the cells with splicing inhibitor madrasin led to similar loss of POLR2A and reduction of nascent transcripts. Although it should be noted that madrasin treatment also led to decreased XAB2 level and thus the observed effect of madrasin on POLR2A might be partially due to reduced XAB2 activity.

It has been documented that nuclear speckles may act as assembly/modification/storage/recycling compartments that can provide splicing factors to active transcription sites (53,54), and inhibition of transcription (55) or pre-mRNA splicing (50,56,57) results in rounded and enlarged nuclear speckles called ‘mega-speckles’. Interestingly, our IF staining also showed that nuclear speckles were partially altered to rounded and enlarged shape in XAB2-depleted or madrasin treatment cells, indicating that XAB2 deficiency may induce transcription or pre-mRNA splicing blockage. However, SC35 staining was not exactly the same from what was observed for XAB2 depletion and madrasin treatment, XAB2 knockdown also caused collapsed SC35 dots and nuclei, which might have been due to mitotic defects, and madrasin treatment also resulted in diffuse SC35 staining.

It is surprising to mention that inhibition of translation can restore POLR2A mRNA level, whereas depletion of Dom34 restores POLR2A at both RNA and protein levels, this effect is due to increased stability of POLR2A mRNA. Thus in addition to the splicing defects in POLR2A induced by XAB2 deficiency, the level of Dom34 and other proteins in mRNA surveillance may also have an impact on POLR2A mRNA expression. Previous studies revealed that Dom34 and HBS1 joined exosome-Ski complex to function in non-stop decay (58) and no-go decay (59), our finding implies that Dom34 may also play a critical role in the degradation of correctly processed mRNA, it will be interesting to investigate what is the difference between complexes for normal mRNA degradation and for aberrant RNA decay.

Our previous data showed that XAB2 knockdown led to cell cycle arrest and DNA damage (31), XAB2 was also reported to be down-regulated dramatically in aged hematopoietic stem cells by microarray analysis (60), suggesting that XAB2 may regulate cellular senescence. In addition, expression of POLR2A is significantly decreased in Werner syndrome patients or old human donor cells compared with young donor cells, also implying a role in cellular senescence (12). Therefore, we tested the possibility that XAB2 regulates cellular senescence via POLR2A. Indeed, SA-β-gal staining indicated that both XAB2 and POLR2A depletion led to dramatic cellular senescence in human skin fibroblast HFF1 cells. Western blot further showed that the expression of p53 and p21 was apparently increased after XAB2 knockdown. This observation is consistent with previous studies that senescence induced by DNA-damage is usually manifested as activated p53 and its downstream target p21 (61,62). Strikingly, XAB2 knockdown followed by POLR2A over-expression rescued the senescent phenotype caused by XAB2 depletion, which supports POLR2A as the key mediator of cell senescence induced by XAB2 depletion. Since cellular senescence has been associated with aging and age-related diseases (63), it will be interesting to investigate whether XAB2/POLR2A pathway can serve as new markers or therapy targets for aging or age-related diseases.

**Figure 8.**
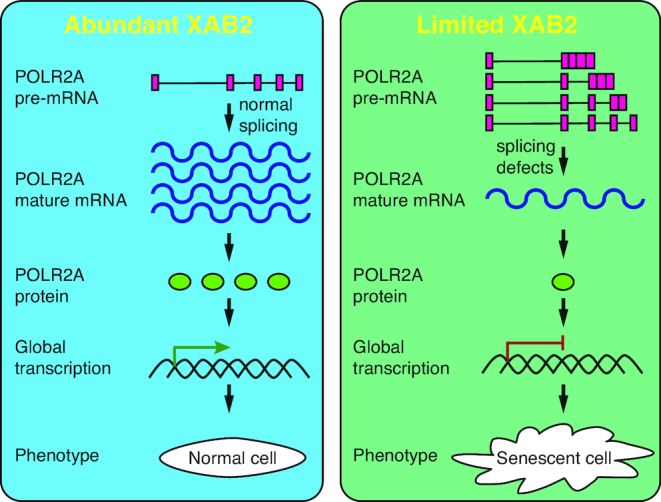
Model for XAB2 in gene expression and cellular senescence.

Based on these data, we propose a model for the role of XAB2 in gene expression (Figure 8). At the deficiency of XAB2, splicing of many genes including POLR2A goes wrong which leads to reduction of POLR2A. Loss of POLR2A triggers deregulation of overall gene expression and promotes senescent phenotype, so abundant XAB2 is critical in maintaining correct splicing of POLR2A to govern global gene expression and antagonize cell senescence.

While the current study expands our understanding of how XAB2 is involved in gene expression, it raises more interesting questions for future investigation. Detailed function of XAB2 in splicing remains to be dissected, common features of introns with retention are still elusive. Further study will also be needed to gain deep insight into how Dom34 or other proteins in mRNA surveillance function in POLR2A mRNA degradation. Finally, it is fascinating to explore whether POLR2A plays a more general role in different types of senescence or causes human diseases at its deficiency.

## DATA AVAILABILITY

RNA-seq data have been deposited at the NCBI Gene Expression Omnibus under the accession number GSE130087.

## Supplementary Material

gkz532_Supplemental_Files

## References

[1] Komili S., Silver P.A. Coupling and coordination in gene expression processes: a systems biology view. Nat. Rev. Genet. 2008; 9:38–48. 18071322 10.1038/nrg2223

[2] Hernandez-Segura A., Nehme J., Demaria M. Hallmarks of cellular senescence. Trends Cell Biol. 2018; 28:436–453. 29477613 10.1016/j.tcb.2018.02.001

[3] Smith Z.D., Sindhu C., Meissner A. Molecular features of cellular reprogramming and development. Nat. Rev. Mol. Cell Biol. 2016; 17:139–154. 26883001 10.1038/nrm.2016.6

[4] Henning A.N., Roychoudhuri R., Restifo N.P. Epigenetic control of CD8(+) T cell differentiation. Nat. Rev. Immunol. 2018; 18:340–356. 29379213 10.1038/nri.2017.146 PMC6327307

[5] de Nadal E., Ammerer G., Posas F. Controlling gene expression in response to stress. Nat. Rev. Genet. 2011; 12:833–845. 22048664 10.1038/nrg3055

[6] Rue P., Arias A.M. Cell dynamics and gene expression control in tissue homeostasis and development. Mol. Syst. Biol. 2015; 11:792. 25716053 10.15252/msb.20145549 PMC4358661

[7] Carpenter S., Ricci E.P., Mercier B.C., Moore M.J., Fitzgerald K.A. Post-transcriptional regulation of gene expression in innate immunity. Nat. Rev. Immunol. 2014; 14:361–376. 24854588 10.1038/nri3682

[8] Jeronimo C., Bataille A.R., Robert F. The writers, readers, and functions of the RNA polymerase II C-terminal domain code. Chem. Rev. 2013; 113:8491–8522. 23837720 10.1021/cr4001397

[9] Jeronimo C., Collin P., Robert F. The RNA polymerase II CTD: the increasing complexity of a low-complexity protein domain. J. Mol. Biol. 2016; 428:2607–2622. 26876604 10.1016/j.jmb.2016.02.006

[10] Eick D., Geyer M. The RNA polymerase II carboxy-terminal domain (CTD) code. Chem. Rev. 2013; 113:8456–8490. 23952966 10.1021/cr400071f

[11] Saldi T., Cortazar M.A., Sheridan R.M., Bentley D.L. Coupling of RNA polymerase II transcription elongation with Pre-mRNA splicing. J. Mol. Biol. 2016; 428:2623–2635. 27107644 10.1016/j.jmb.2016.04.017 PMC4893998

[12] Kyng K.J., May A., Kolvraa S., Bohr V.A. Gene expression profiling in Werner syndrome closely resembles that of normal aging. Proc. Natl. Acad. Sci. U.S.A. 2003; 100:12259–12264. 14527998 10.1073/pnas.2130723100 PMC218746

[13] Clarke V.E., Harmanci A.S., Bai H.W., Youngblood M.W., Lee T.I., Baranoski J.F., Ercan-Sencicek A.G., Abraham B.J., Weintraub A.S., Hnisz D. et al. Recurrent somatic mutations in POLR2A define a distinct subset of meningiomas. Nat. Genet. 2016; 48:1253–1259. 27548314 10.1038/ng.3651 PMC5114141

[14] Anindya R., Aygun O., Svejstrup J.Q. Damage-induced ubiquitylation of human RNA polymerase II by the ubiquitin ligase Nedd4, but not Cockayne syndrome proteins or BRCA1. Mol. Cell. 2007; 28:386–397. 17996703 10.1016/j.molcel.2007.10.008

[15] Yasukawa T., Kamura T., Kitajima S., Conaway R.C., Conaway J.W., Aso T. Mammalian Elongin A complex mediates DNA-damage-induced ubiquitylation and degradation of Rpb1. EMBO J. 2008; 27:3256–3266. 19037258 10.1038/emboj.2008.249 PMC2609743

[16] Kleiman F.E., Wu-Baer F., Fonseca D., Kaneko S., Baer R., Manley J.L. BRCA1/BARD1 inhibition of mRNA 3′ processing involves targeted degradation of RNA polymerase II. Gene Dev. 2005; 19:1227–1237. 15905410 10.1101/gad.1309505 PMC1132008

[17] Karakasili E., Burkert-Kautzsch C., Kieser A., Strasser K. Degradation of DNA damage-independently stalled RNA polymerase II is independent of the E3 ligase Elc1. Nucleic Acids Res. 2014; 42:10503–10515. 25120264 10.1093/nar/gku731 PMC4176355

[18] Wilson M.D., Harreman M., Svejstrup J.Q. Ubiquitylation and degradation of elongating RNA polymerase II: The last resort. Bba-Gene Regul. Mech. 2013; 1829:151–157. 10.1016/j.bbagrm.2012.08.002 22960598

[19] Somesh B.P., Reid J., Liu W.F., Sogaard T.M.M., Erdjument-Bromage H., Tempst P., Svejstrup J.Q. Multiple mechanisms confining RNA polymerase II ubiquitylation to polymerases undergoing transcriptional arrest. Cell. 2005; 121:913–923. 15960978 10.1016/j.cell.2005.04.010

[20] Shi Y.G. Mechanistic insights into precursor messenger RNA splicing by the spliceosome. Nat. Rev. Mol. Cell Biol. 2017; 18:655–670. 28951565 10.1038/nrm.2017.86

[21] Will C.L., Luhrmann R. Spliceosome structure and function. Csh. Perspect. Biol. 2011; 3:a003707. 10.1101/cshperspect.a003707 PMC3119917 21441581

[22] Wahl M.C., Will C.L., Luhrmann R. The spliceosome: design principles of a dynamic RNP machine. Cell. 2009; 136:701–718. 19239890 10.1016/j.cell.2009.02.009

[23] Shi Y.G. The spliceosome: a protein-directed metalloribozyme. J. Mol. Biol. 2017; 429:2640–2653. 28733144 10.1016/j.jmb.2017.07.010

[24] Faustino N.A., Cooper T.A. Pre-mRNA splicing and human disease. Gene Dev. 2003; 17:419–437. 12600935 10.1101/gad.1048803

[25] Poulos M.G., Batra R., Charizanis K., Swanson M.S. Developments in RNA splicing and disease. Csh. Perspect. Biol. 2011; 3:a000778. 10.1101/cshperspect.a000778 PMC3003463 21084389

[26] Singh R.K., Cooper T.A. Pre-mRNA splicing in disease and therapeutics. Trends Mol. Med. 2012; 18:472–482. 22819011 10.1016/j.molmed.2012.06.006 PMC3411911

[27] Nakatsu Y., Asahina H., Citterio E., Rademakers S., Vermeulen W., Kamiuchi S., Yeo J.P., Khaw M.C., Saijo M., Kodo N. et al. XAB2, a novel tetratricopeptide repeat protein involved in transcription-coupled DNA repair and transcription. J. Biol. Chem. 2000; 275:34931–34937. 10944529 10.1074/jbc.M004936200

[28] Kuraoka I., Ito S., Wada T., Hayashida M., Lee L., Saijo M., Nakatsu Y., Matsumoto M., Matsunaga T., Handa H. et al. Isolation of XAB2 complex involved in pre-mRNA splicing, transcription, and transcription-coupled repair. J. Biol. Chem. 2008; 283:940–950. 17981804 10.1074/jbc.M706647200

[29] Onyango D.O., Howard S.M., Neherin K., Yanez D.A., Stark J.M. Tetratricopeptide repeat factor XAB2 mediates the end resection step of homologous recombination. Nucleic. Acids. Res. 2016; 44:5702–5716. 27084940 10.1093/nar/gkw275 PMC4937314

[30] Lei H., Zhai B., Yin S., Gygi S., Reed R. Evidence that a consensus element found in naturally intronless mRNAs promotes mRNA export. Nucleic Acids Res. 2013; 41:2517–2525. 23275560 10.1093/nar/gks1314 PMC3575797

[31] Hou S., Li N., Zhang Q., Li H., Wei X.Y., Hao T., Li Y., Azam S., Liu C.G., Cheng W. et al. XAB2 functions in mitotic cell cycle progression via transcriptional regulation of CENPE. Cell Death Dis. 2016; 7:e2409. 27735937 10.1038/cddis.2016.313 PMC5133980

[32] De I., Sessonov S., Hofele R., dos Santos K., Will C.L., Urlaub H., Luhrmann R., Pena V. The RNA helicase Aquarius exhibits structural adaptations mediating its recruitment to spliceosomes. Nat. Struct. Mol. Biol. 2015; 22:138–144. 25599396 10.1038/nsmb.2951

[33] Deckert J., Hartmuth K., Boehringer D., Behzadnia N., Will C.L., Kastner B., Stark H., Urlaub H., Luhrmann R. Protein composition and electron microscopy structure of affinity-purified human spliceosomal B complexes isolated under physiological conditions. Mol. Cell. Biol. 2006; 26:5528–5543. 16809785 10.1128/MCB.00582-06 PMC1592722

[34] Bessonov S., Anokhina M., Krasauskas A., Golas M.M., Sander B., Will C.L., Urlaub H., Stark H., Luhrmann R. Characterization of purified human B act spliceosomal complexes reveals compositional and morphological changes during spliceosome activation and first step catalysis. RNA. 2010; 16:2384–2403. 20980672 10.1261/rna.2456210 PMC2995400

[35] Haselbach D., Komarov I., Agafonov D.E., Hartmuth K., Graf B., Dybkov O., Urlaub H., Kastner B., Luhrmann R., Stark H. Structure and conformational dynamics of the human spliceosomal B-act complex. Cell. 2018; 172:454–464. 29361316 10.1016/j.cell.2018.01.010

[36] Zhang X.F., Yan C.Y., Zhan X.C., Li L.J., Lei J.L., Shi Y.G. Structure of the human activated spliceosome in three conformational states. Cell Res. 2018; 28:307–322. 29360106 10.1038/cr.2018.14 PMC5835773

[37] Zhan X.C., Yan C.Y., Zhang X.F., Lei J.L., Shi Y.G. Structure of a human catalytic step I spliceosome. Science. 2018; 359:537–544. 29301961 10.1126/science.aar6401

[38] Bertram K., Agafonov D.E., Liu W.T., Dybkov O., Will C.L., Hartmuth K., Urlaub H., Kastner B., Stark H., Luhrmann R. Cryo-EM structure of a human spliceosome activated for step 2 of splicing. Nature. 2017; 542:318–323. 28076346 10.1038/nature21079

[39] Zhang X.F., Yan C.Y., Hang J., Finci L.I., Lei J.L., Shi Y.G. An atomic structure of the human spliceosome. Cell. 2017; 169:918–929. 28502770 10.1016/j.cell.2017.04.033

[40] Russell C.S., Ben-Yehuda S., Dix I., Kupiec M., Beggs J.D. Functional analyses of interacting factors involved in both pre-mRNA splicing and cell cycle progression in Saccharomyces cerevisiae. RNA. 2000; 6:1565–1572. 11105756 10.1017/s1355838200000984 PMC1370026

[41] Chanarat S., Seizl M., Strasser K. The Prp19 complex is a novel transcription elongation factor required for TREX occupancy at transcribed genes. Gene Dev. 2011; 25:1147–1158. 21576257 10.1101/gad.623411 PMC3110953

[42] Minocha R., Popova V., Kopytova D., Misiak D., Huttelmaier S., Georgieva S., StraSser K. Mud2 functions in transcription by recruiting the Prp19 and TREX complexes to transcribed genes. Nucleic Acids Res. 2018; 46:9749–9763. 30053068 10.1093/nar/gky640 PMC6182176

[43] Robinson J.T., Thorvaldsdottir H., Winckler W., Guttman M., Lander E.S., Getz G., Mesirov J.P. Integrative genomics viewer. Nat. Biotechnol. 2011; 29:24–26. 21221095 10.1038/nbt.1754 PMC3346182

[44] Wang Q.Q., Rio D.C. JUM is a computational method for comprehensive annotation-free analysis of alternative pre-mRNA splicing patterns. Proc. Natl. Acad. Sci. U.S.A. 2018; 115:Eb181–Eb190. 10.1073/pnas.1806018115 PMC6126775 30104386

[45] Folco E.G., Lei H.X., Hsu J.L., Reed R. Small-scale nuclear extracts for functional assays of gene-expression machineries. Jove-J. Vis. Exp. 2012; 64:4140. 10.3791/4140 PMC3471294 22782264

[46] Perez-Riverol Y., Csordas A., Bai J.W., Bernal-Llinares M., Hewapathirana S., Kundu D.J., Inuganti A., Griss J., Mayer G., Eisenacher M. et al. The PRIDE database and related tools and resources in 2019: improving support for quantification data. Nucleic Acids Res. 2019; 47:D442–D450. 30395289 10.1093/nar/gky1106 PMC6323896

[47] Chen E.Y., Tan C.M., Kou Y., Duan Q.N., Wang Z.C., Meirelles G.V., Clark N.R., Ma’ayan A. Enrichr: interactive and collaborative HTML5 gene list enrichment analysis tool. BMC Bioinformatics. 2013; 14:128. 23586463 10.1186/1471-2105-14-128 PMC3637064

[48] Kuleshov M.V., Jones M.R., Rouillard A.D., Fernandez N.F., Duan Q.N., Wang Z.C., Koplev S., Jenkins S.L., Jagodnik K.M., Lachmann A. et al. Enrichr: a comprehensive gene set enrichment analysis web server 2016 update. Nucleic Acids Res. 2016; 44:W90–W97. 27141961 10.1093/nar/gkw377 PMC4987924

[49] Steckelberg A.L., Altmueller J., Dieterich C., Gehring N.H. CWC22-dependent pre-mRNA splicing and eIF4A3 binding enables global deposition of exon junction complexes. Nucleic Acids Res. 2015; 43:4687–4700. 25870412 10.1093/nar/gkv320 PMC4482076

[50] Tanackovic G., Kramer A. Human splicing factor SF3a, but not SF1, is essential for pre-mRNA splicing in vivo. Mol. Biol. Cell. 2005; 16:1366–1377. 15647371 10.1091/mbc.E04-11-1034 PMC551499

[51] Ji X., Zhou Y., Pandit S., Huang J.E., Li H.R., Lin C.Y., Xiao R., Burge C.B., Fu X.D. SR proteins collaborate with 7SK and promoter-associated nascent RNA to release paused polymerase. Cell. 2013; 153:855–868. 23663783 10.1016/j.cell.2013.04.028 PMC4103662

[52] Koga M., Hayashi M., Kaida D. Splicing inhibition decreases phosphorylation level of Ser2 in Pol II CTD. Nucleic Acids Res. 2015; 43:8258–8267. 26202968 10.1093/nar/gkv740 PMC4787822

[53] Lamond A.I., Spector D.L. Nuclear speckles: a model for nuclear organelles. Nat. Rev. Mol. Cell Biol. 2003; 4:605–612. 12923522 10.1038/nrm1172

[54] Spector D.L., Lamond A.I. Nuclear speckles. Csh Perspect. Biol. 2011; 3:a000646. 10.1101/cshperspect.a000646 PMC3039535 20926517

[55] Lallena M.J., Correas I. Transcription-dependent redistribution of nuclear protein 4.1 to SC35-enriched nuclear domains. J. Cell Sci. 1997; 110:239–247. 9044054 10.1242/jcs.110.2.239

[56] Effenberger K.A., Anderson D.D., Bray W.M., Prichard B.E., Ma N.C., Adams M.S., Ghosh A.K., Jurica M.S. Coherence between cellular responses and in vitro splicing inhibition for the anti-tumor drug pladienolide B and its analogs. J. Biol. Chem. 2014; 289:1938–1947. 24302718 10.1074/jbc.M113.515536 PMC3900944

[57] Onyango D.O., Lee G., Stark J.M. PRPF8 is important for BRCA1-mediated homologous recombination. Oncotarget. 2017; 8:93319–93337. 29212152 10.18632/oncotarget.21555 PMC5706798

[58] Saito S., Hosoda N., Hoshino S. The Hbs1-Dom34 protein complex functions in non-stop mRNA decay in mammalian cells. J. Biol. Chem. 2013; 288:17832–17843. 23667253 10.1074/jbc.M112.448977 PMC3682582

[59] Shoemaker C.J., Eyler D.E., Green R. Dom34:Hbs1 promotes subunit dissociation and peptidyl-tRNA drop-off to initiate No-Go decay. Science. 2010; 330:369–372. 20947765 10.1126/science.1192430 PMC4022135

[60] Chambers S.M., Shaw C.A., Gatza C., Fisk C.J., Donehower L.A., Goodell M.A. Aging hematopoietic stem cells decline in function and exhibit epigenetic dysregulation. PLoS Biol. 2007; 5:1750–1762. 10.1371/journal.pbio.0050201 PMC1925137 17676974

[61] Munoz-Espin D., Serrano M. Cellular senescence: from physiology to pathology. Nat. Rev. Mol. Cell Biol. 2014; 15:482–496. 24954210 10.1038/nrm3823

[62] Campisi J., di Fagagna F.D. Cellular senescence: when bad things happen to good cells. Nat. Rev. Mol. Cell Biol. 2007; 8:729–740. 17667954 10.1038/nrm2233

[63] Childs B.G., Gluscevic M., Baker D.J., Laberge R.M., Marquess D., Dananberg J., van Deursen J.M. Senescent cells: an emerging target for diseases of ageing. Nat. Rev. Drug Discov. 2017; 16:718–735. 28729727 10.1038/nrd.2017.116 PMC5942225

