# Supplementary material for: XAB2 depletion induces intron retention in POLR2A to impair global transcription and promote cellular senescence
Source: Nucleic Acids Res. 2019 Jun 19;47(15):8239–54. doi: 10.1093/nar/gkz532 (PMC6735682; doi:10.1093/nar/gkz532)
Supplement: gkz532_Supplemental_Files [file gkz532_supplemental_files.zip › SUPPLEMENTARY DATA 2019-6-1.docx]

**SUPPLEMENTARY DATA**

**MATERIALS AND METHODS**

**Chromatin-associated nascent RNA isolation**

Cellular fractionation and chromatin-associated nascent RNA isolation was performed as previously described with some modifications ([1-3](#_ENREF_1)). Briefly, 1x10^7^ HeLa cells with XAB2 knockdown using Dox-inducible system were trypsinized and collected by centrifugation at 500 g at 4 °C for 5 min. The cell pellets were then washed once with 5 mL ice-cold PBS, resuspended in 2 mL ice-cold HLB+N buffer (10 mM Tris-HCl, pH 7.5, 10 mM NaCl, 2.5 mM MgCl_2_, 0.5% (vol/vol) NP-40), and incubated on ice for 5 min. Cellular lysates were passed through 0.5 mL ice-cold HLB+NS buffer (10 mM Tris-HCl, pH 7.5, 10 mM NaCl, 2.5 mM MgCl_2_, 0.5% (vol/vol) NP-40, 10% (wt/vol) sucrose) at 500 g at 4 °C for 5 min. The nuclear pellets were resuspended in 62.5 uL ice-cold NUN1 buffer (20 mM Tris-HCl, pH 7.9, 75 mM NaCl, 0.5 mM EDTA and 50% (vol/vol) glycerol), added with 0.6 mL ice-cold NUN2 buffer (20 mM HEPES-KOH, pH 7.6, 300 mM NaCl, 0.2 mM EDTA, 7.5 mM MgCl_2_, 1% (vol/vol) NP-40, 1 M urea), vortexed for 4 seconds, incubated on ice for 2 min, and then centrifuged at 16,000 g at 4 °C for 2 min. The chromatin pellet was rinsed with 1 mL PBS and then dissolved in 1 mL TRIzol (Sigma). Nascent RNA was purified according to the TRIzol protocol. 1 ug total RNA was treated with DNase I and then analyzed by RT-PCR.

**ChIP**

ChIP experiment was performed as previously described with some modifications ([4](#_ENREF_4),[5](#_ENREF_5)). Briefly, HeLa cells with XAB2 knockdown using Dox-inducible system cultured in 10 cm plate were crosslinked with 1% formaldehyde at room temperature for 10 min, quenched with 125 mM glycine for 5 min, washed with PBS for two times. Cells were scraped and then resuspended in IP buffer (66.7 mM Tris-HCl, pH 8.0, 100 mM NaCl, 5 mM EDTA, 0.33% SDS, 1.67% Triton X-100, 0.02% NaN_3_) for sonication to get soluble chromatin. The lysate was diluted with IP buffer, pre-cleared with 20 ul protein A/G plus agarose beads (Santa Cruz, sc-2003) at 4 °C for 30min, followed by incubation with 4 ug POLR2A (8WG16) antibody (Abcam, ab817) or mouse IgG (Santa Cruz, sc-2025) at 4 °C overnight. Immunoprecipitated complexes were captured with 50 ul protein A/G plus agarose beads at 4 °C for 4 h. Beads were washed twice in wash buffer 1 (20 mM Tris-HCl, pH 8.0, 150 mM NaCl, 5 mM EDTA, 0.2% SDS, 1% Triton X-100, 5.2% sucrose, 0.02% NaN_3_), twice in wash buffer 2 (50 mM HEPES, pH 7.5, 500 mM NaCl, 1 mM EDTA, 1% Triton X-100, 0.1% deoxycholic acid, 0.02% NaN_3_), twice in wash buffer 3 (10 mM Tris-HCl, pH 8.0, 250 mM LiCl, 1 mM EDTA, 0.5% NP-40, 0.5% deoxycholic acid, 0.02% NaN_3_), once in TE buffer, and then eluted and reversed crosslinks in 300 ul elution buffer (1% SDS and 0.1 M NaHCO_3_) at 65 °C overnight, followed by treatment with 0.2 mg/mL proteinase K at 37°C for 4 h. Input and eluted DNA was purified with phenol/chloroform/isopentanol and precipitated with ethanol. The pellets were resuspended in water and analyzed by PCR.

**Figure S1. XAB2 depletion led to loss of POLR2A in 293T or MDA-MB-231 cells.**

A. Down-regulation of POLR2A at RNA level after XAB2 depletion in 293T or MDA-MB-231 cells. Cells were harvested after 48 hours of siRNA treatment or 72 hours of shRNA treatment.

B. Reduction of POLR2A protein after XAB2 depletion in 293T or MDA-MB-231 cells. Cells were harvested after 48 hours of siRNA treatment or 72 hours of shRNA treatment.

**Figure S2. Immunofluorescence staining to show substantial loss of POLR2A and altered SC35 pattern after XAB2 depletion.**

**Figure S3. Time points of POLR2A decrease at RNA and protein levels after XAB2 depletion.**

A. POLR2A RNA decreased at 24 h after Dox-induced expression of XAB2 shRNA in HeLa cells.

B. Reduction of POLR2A protein was observed at 72 h after Dox-induced expression of XAB2 shRNA in HeLa cells.

**Figure S4. Decrease of POLR2A mRNA after XAB2 depletion was not due to reduced transcription.**

A. Luciferase assay using POLR2A and CENPE promoter constructs after XAB2 depletion.

B. Reduction of POLR2A protein was observed at 72 h but not 36 h after Dox-induced expression of XAB2 shRNA in HeLa cells.

C. ChIP assay showing the recruitment of POLR2A to its own gene was not impaired upon XAB2 depletion at 36 h and 72 h.

D. Quantitation of relative gene expression compared to control sample in C. (n=3, no significant difference was observed for the enrichment of POLR2A before and after Dox induction)

E. Western blot analysis showing the chromatin-associated nascent RNA fraction (detected by Histone 3) was not contaminated by cytoplasmic fraction (detected by α-tubulin) and nucleoplasmic fraction (detected by U1-70K). Cyt: cytoplasm, Nuc: nucleoplasm, Chr: chromatin.

F. Nascent RNA assay showing POLR2A nascent transcripts were not reduced after XAB2 knockdown at 36 h and 72 h.

G. Quantitation of relative RNA expression compared to control sample in E. (n=3, ***: P < 0.001, no significant difference was observed at other time points)

**Figure S5. XAB2 depletion induced intron retention.**

A. RNA-seq revealed much more reads mapping to intronic regions after XAB2 depletion. **P < 0.01.

B. Schematic diagram to show striking increase of reads mapping to many introns of POLR2A gene after RNA-seq by IGV (black and red numbers) and JUM (red numbers).

C. RT-PCR analysis validated intron retention after XAB2 knockdown.

D. Quantification showing increased ratio of unspliced vs spliced transcripts.

**Figure S6. Immunofluorescence staining to show substantial loss of POLR2A and altered SC35 pattern after madrasin treatment.**

**Figure S7. Treatment of 293T or MDA-MB-231 cells with splicing inhibitor madrasin resulted in reduction of POLR2A at both RNA and protein levels.**

A. Madrasin treatment led to decrease of POLR2A at RNA level. Cells were harvested after madrasin treatment (30 uM) for 24 hours.

B. Loss of POLR2A protein after madrasin treatment. Cells were harvested after madrasin treatment (30 uM) for 24 hours.

**Figure S8. Translation inhibition had no recovery effect on POLR2A protein level after XAB2 depletion.**

A. Translation inhibition by CHX couldn’t rescue POLR2A at protein level after XAB2 depletion. Cells were treated with 50 ug/ml CHX for 24 hours after XAB2 depletion.

B. Translation inhibition by emetine couldn’t rescue POLR2A at protein level after XAB2 depletion. Cells were treated with 10 ug/ml emetine for 24 hours after XAB2 depletion.

**Figure S9. Depletion of UPF1 after XAB2 knockdown couldn’t rescue POLR2A at RNA level.**

Cells were treated with siRNAs specific for XAB2 and UPF1 for 48 hours.

**Figure S10. Identification of factors functioned in POLR2A reduction after XAB2 depletion.**

A. Western blot analysis validated gene expression levels after XAB2 knockdown. (*Correct band of the protein).

B. Depletion of Dom34, ALY, RNPS1 after XAB2 knockdown partially rescued POLR2A at RNA level. Cells were treated with siRNAs specific for XAB2 and other genes for 48 hours.

C. Depletion of Dom34, ALY, RNPS1 after XAB2 knockdown partially rescued POLR2A at protein level. (*Correct band of the protein). Cells were treated with siRNAs specific for XAB2 and other genes for 48 hours.

**Figure S11. XAB2 knockdown resulted in cell cycle arrest and proliferation inhibition in HFF1 cells.**

A. Depletion of XAB2 caused accumulation of G2/M cells by FACS analysis. HFF1 cells were transfected with siRNAs for 96 hours and then stained with PI for analysis of cell cycle distribution.

B. MTT assay revealed that XAB2 knockdown caused inhibition of cell proliferation (n=3, *P < 0.05, **P < 0.01, ***P < 0.001).

References

1. Nojima, T., Gomes, T., Carmo-Fonseca, M. and Proudfoot, N.J. (2016) Mammalian NET-seq analysis defines nascent RNA profiles and associated RNA processing genome-wide. *Nat Protoc*, **11**, 413-428.

2. Conrad, T. and Orom, U.A. (2017) Cellular Fractionation and Isolation of Chromatin-Associated RNA. *Methods Mol Biol*, **1468**, 1-9.

3. Pandya-Jones, A. and Black, D.L. (2009) Co-transcriptional splicing of constitutive and alternative exons. *RNA*, **15**, 1896-1908.

4. Chen, S.L., Wang, R.J., Zheng, D.H., Zhang, H., Chang, X.Y., Wang, K., Li, W.C., Fan, J., Tian, B. and Cheng, H. (2019) The mRNA Export Receptor NXF1 Coordinates Transcriptional Dynamics, Alternative Polyadenylation, and mRNA Export. *Mol Cell*, **74**, 118-131.

5. Hao, J.J., Xu, H., Luo, M.H., Yu, W.D., Chen, M., Liao, Y.N., Zhang, C.L., Zhao, X.R., Jiang, W., Hou, S. *et al.* (2018) The Tumor-Promoting Role of TRIP4 in Melanoma Progression and its Involvement in Response to BRAF-Targeted Therapy. *J Invest Dermatol*, **138**, 159-170.
