# Supplementary figures and images for: XAB2 depletion induces intron retention in POLR2A to impair global transcription and promote cellular senescence
Source: Nucleic Acids Res. 2019 Jun 19;47(15):8239–54. doi: 10.1093/nar/gkz532 (PMC6735682; doi:10.1093/nar/gkz532)

Fig. S1

A

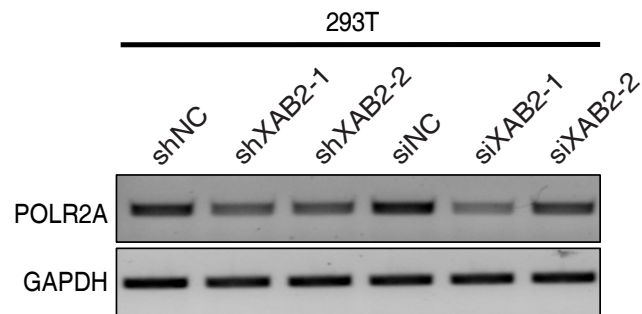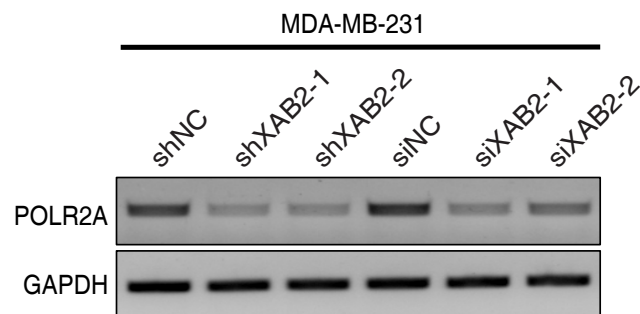

B

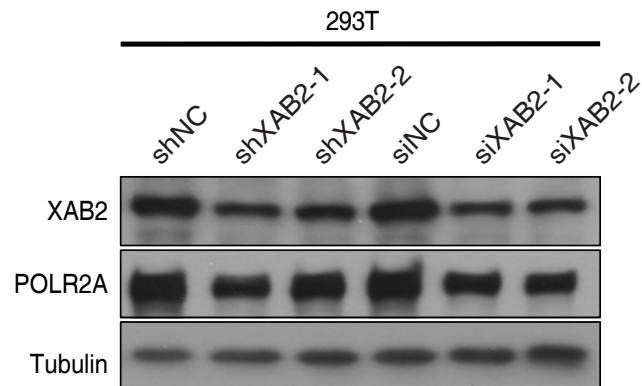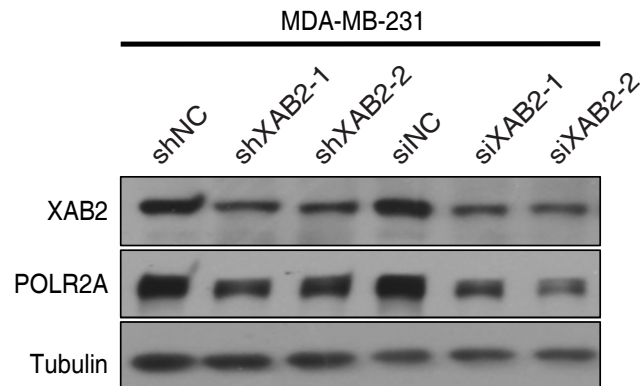

Fig. S2

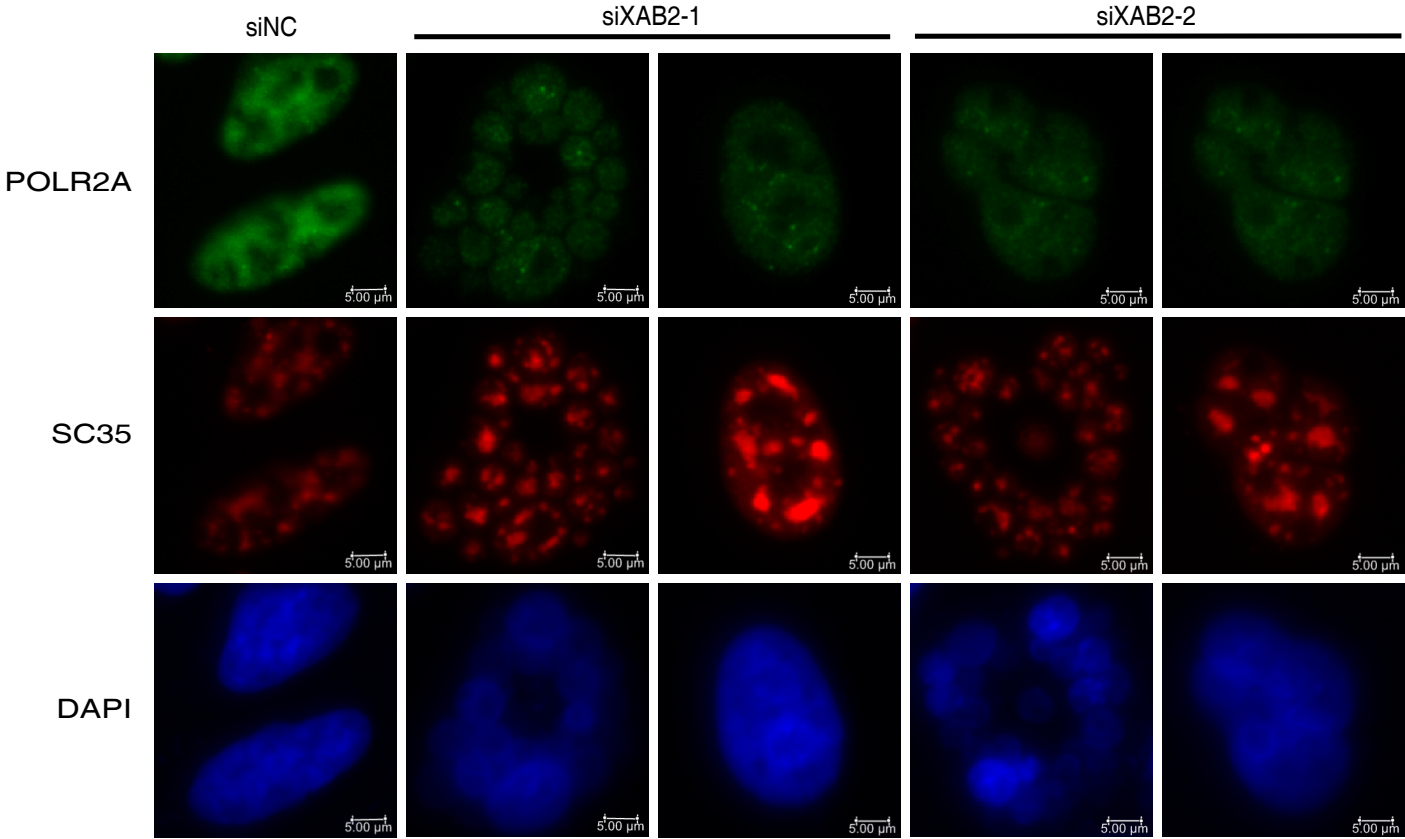

Fig. S3

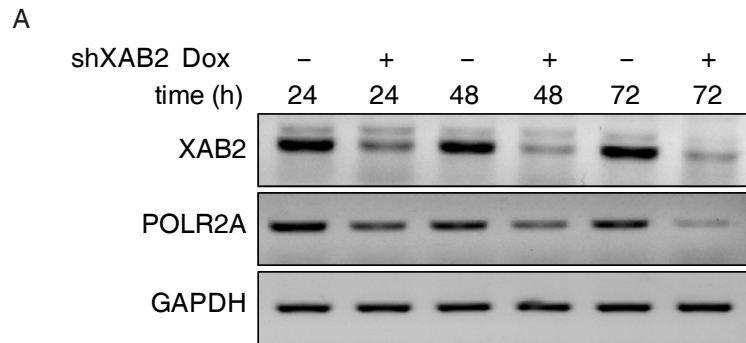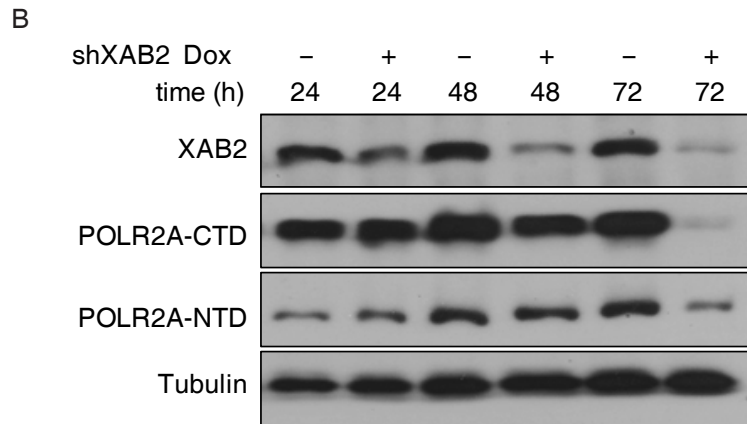

Fig. S4

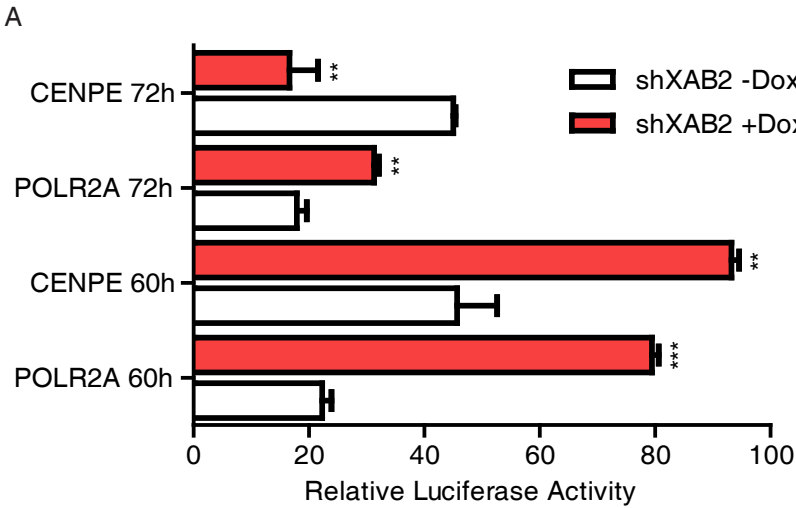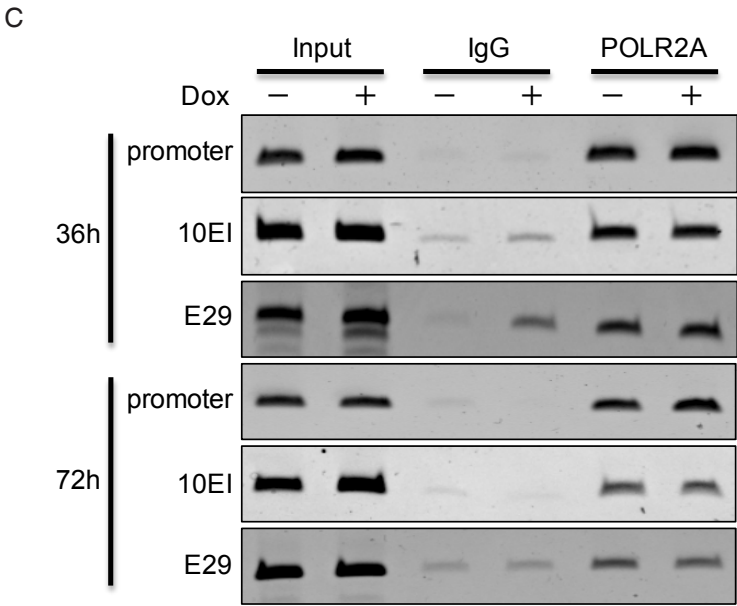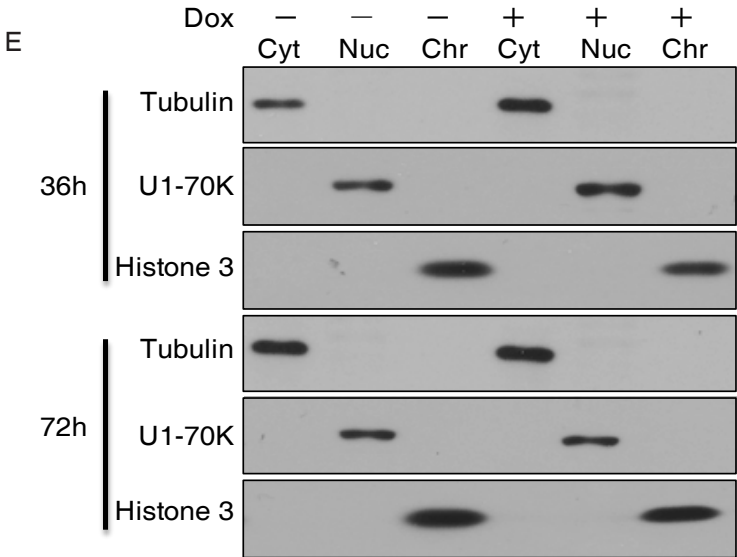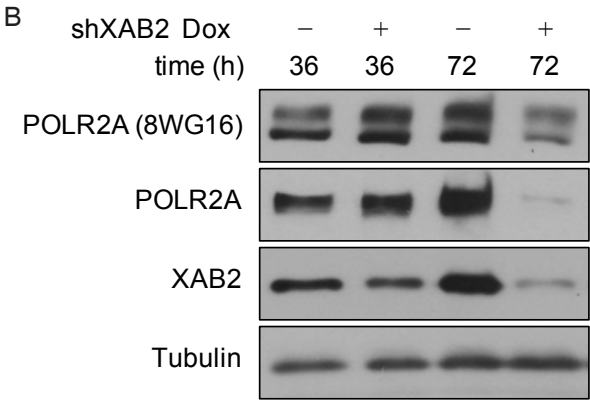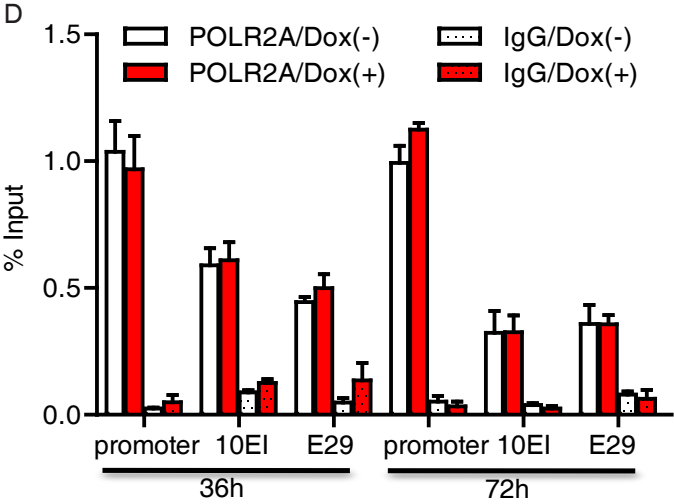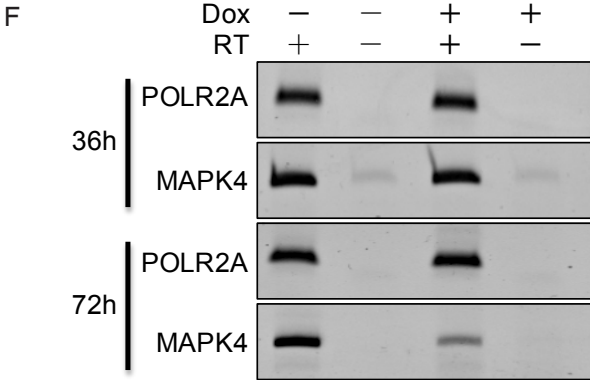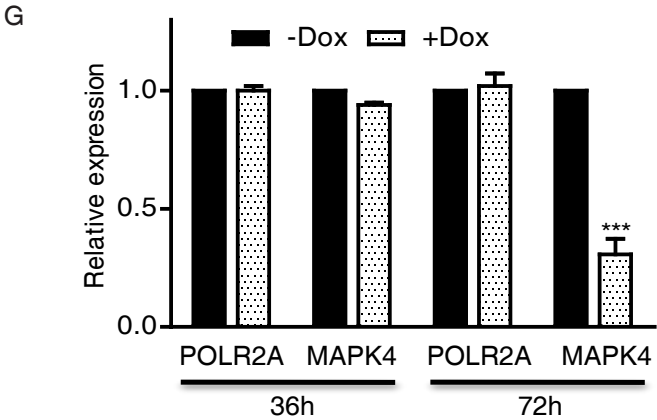

Fig. S5

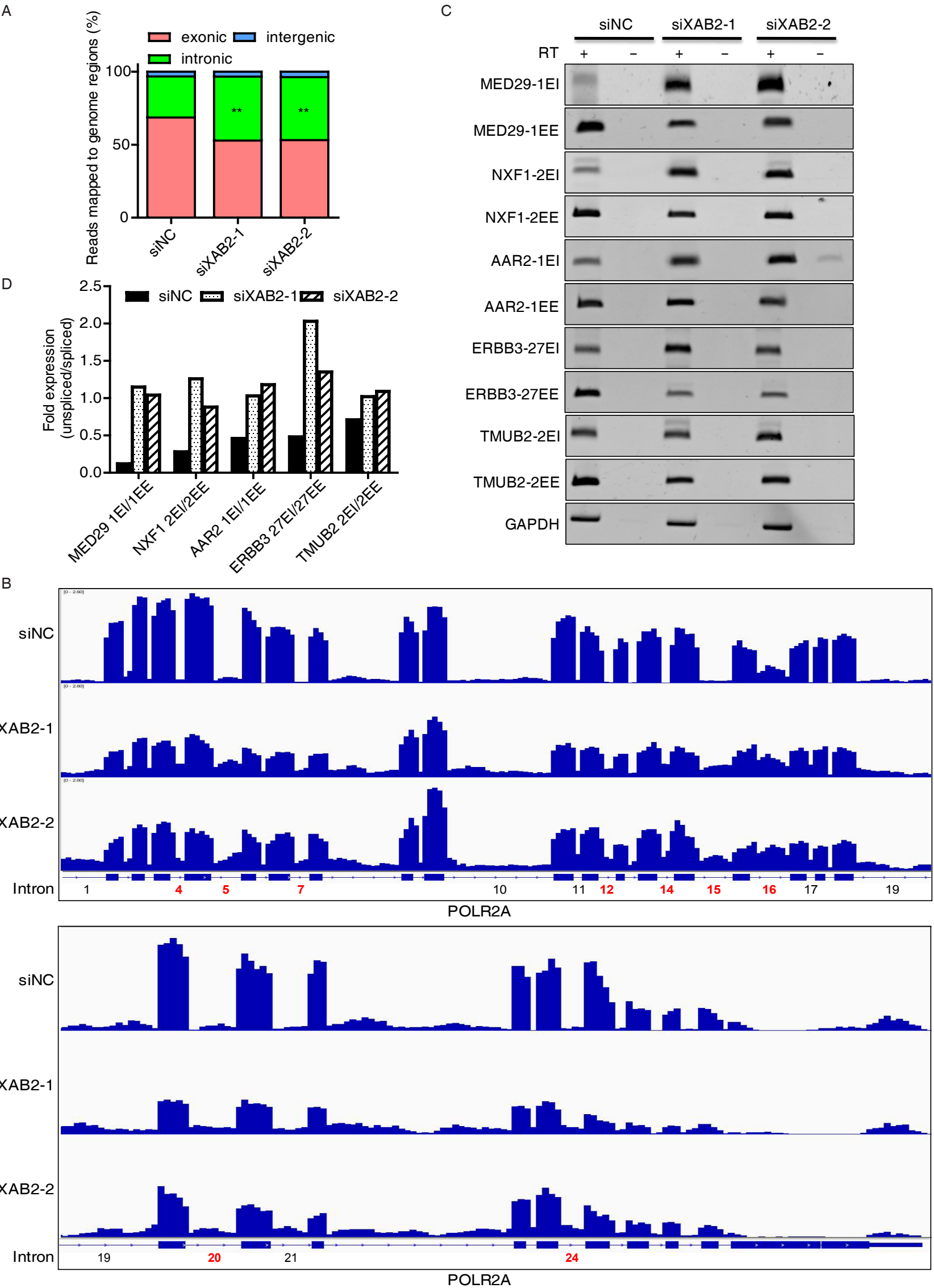

Fig. S6

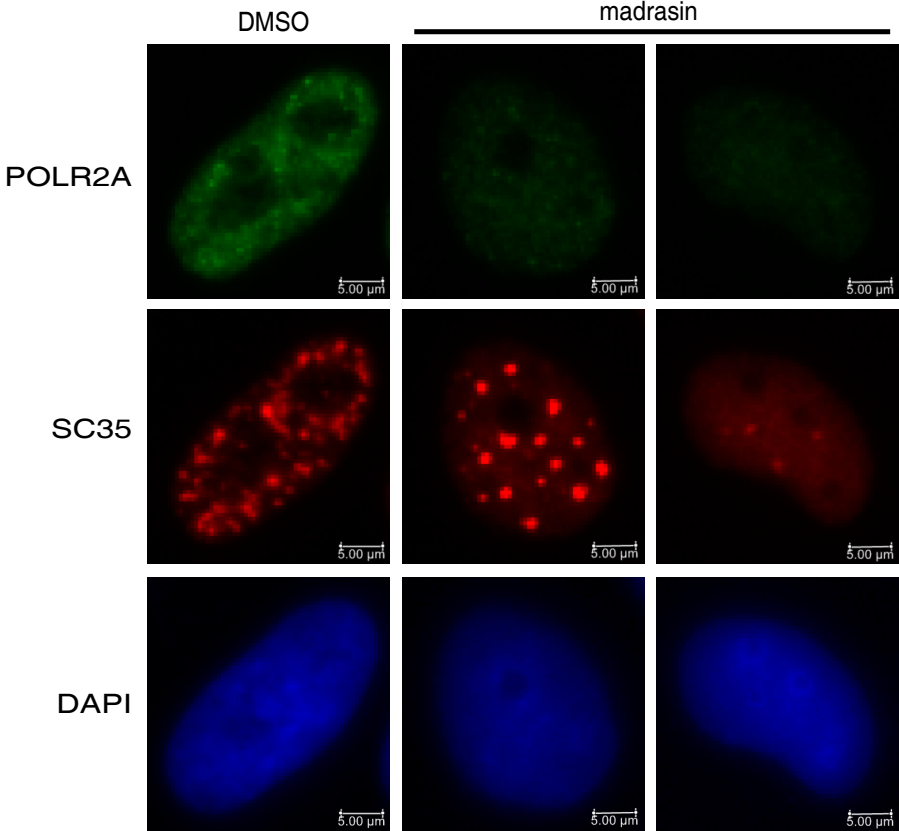

Fig. S7

A

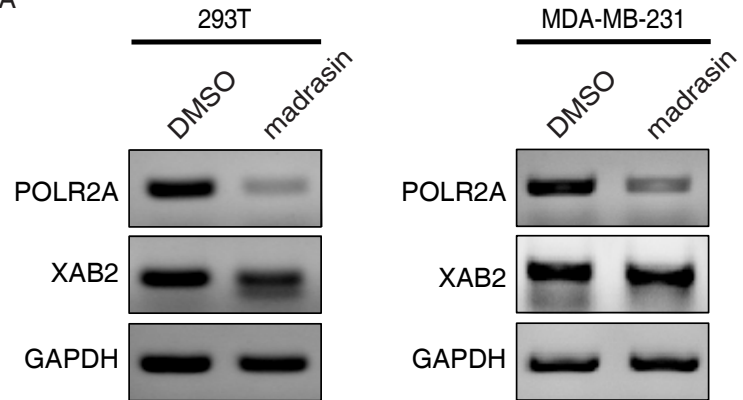

B

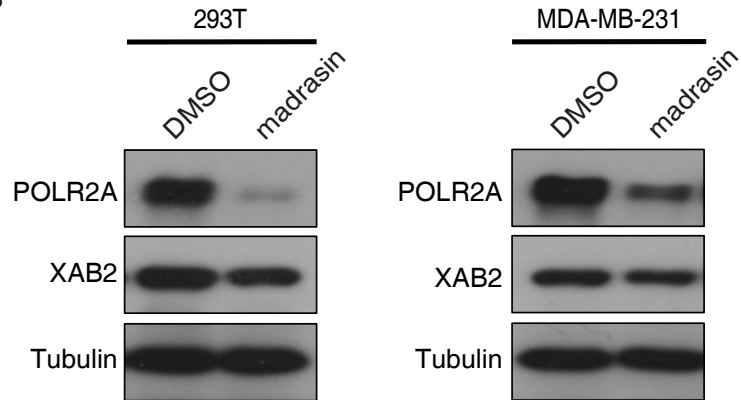

Fig. S8

A

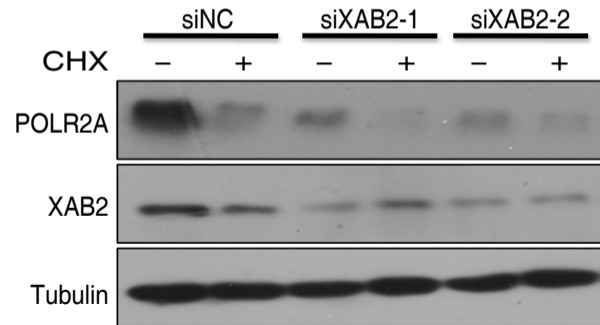

B

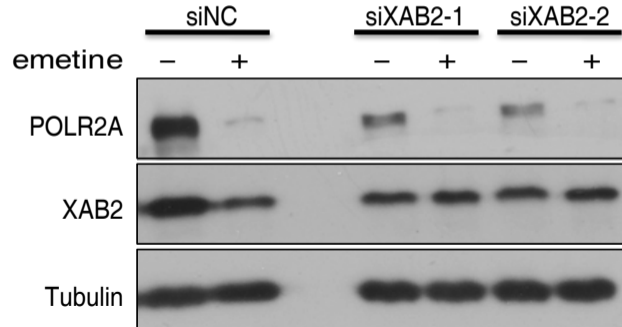

Fig. S9

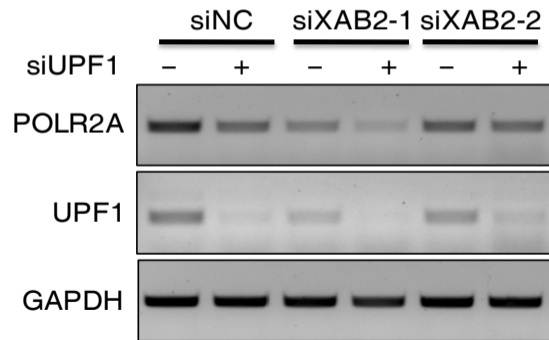

Fig. S10

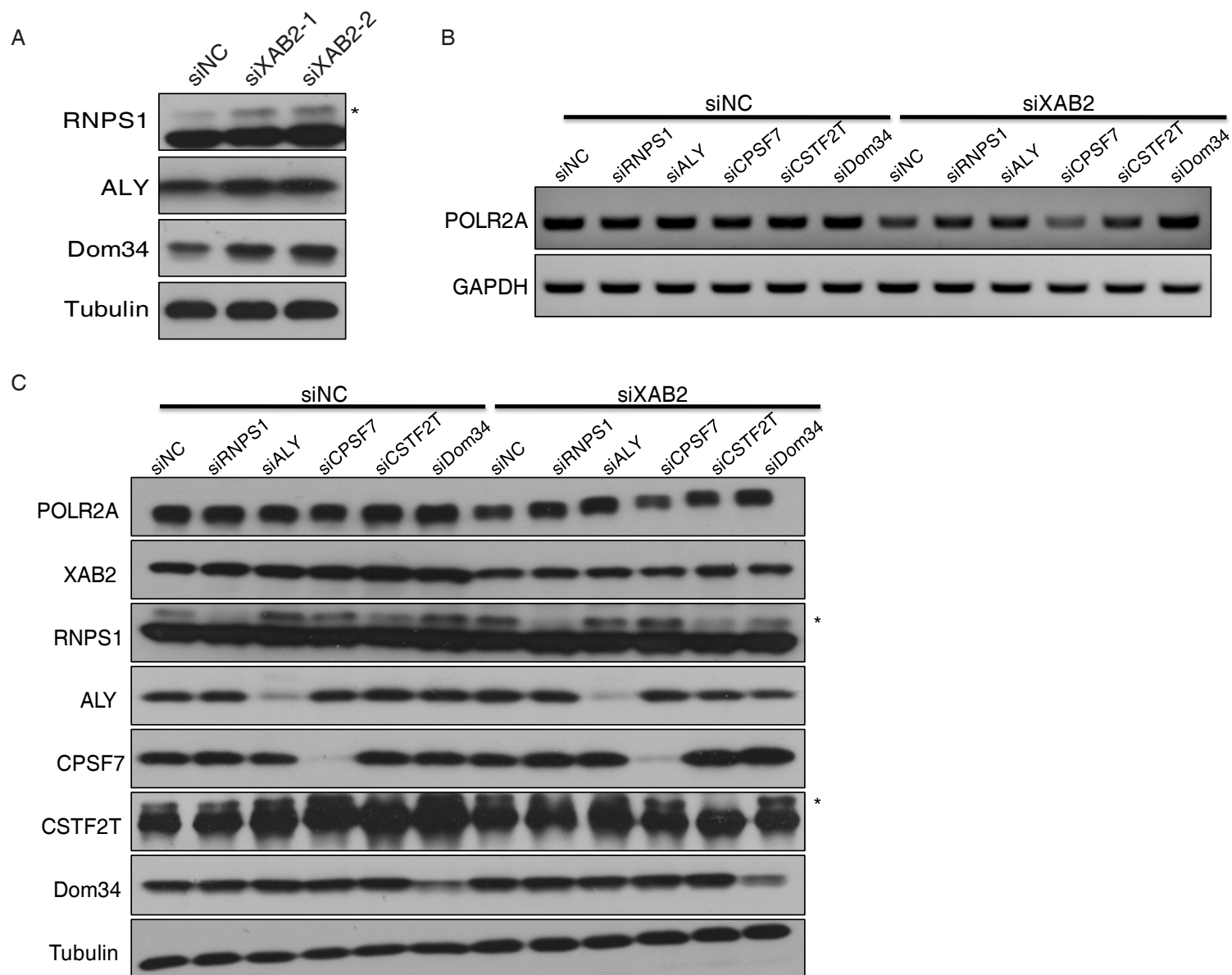

Fig. S11

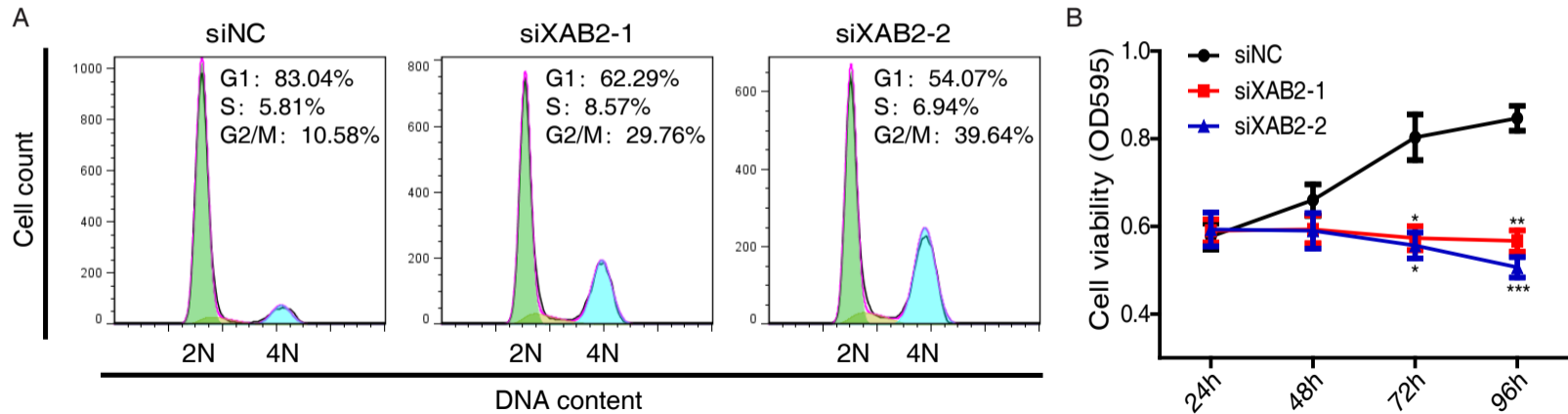

Supplement: gkz532_Supplemental_Files [file gkz532_supplemental_files.zip › suppl fig.pdf]
